# Influence of Reactor Configuration and Operating Conditions on Nanostructured Semiconductor Photocatalysts for Hydrogen Evolution: A Systematic Technical Review

**DOI:** 10.3390/nano16150956

**Published:** 2026-08-03

**Authors:** Jessica Hernández Galván, Luis Angel Iturralde Carrera, Carlos D. Constantino-Robles, Yoisdel Castillo Alvarez, Juvenal Rodríguez-Reséndiz, Rufino Nava

**Affiliations:** 1 Faculty of Chemistry, Autonomous University of Querétaro, Querétaro 76010, Mexico; jhernandez53@alumnos.uaq.mx; 2 Faculty of Engineering, Autonomous University of Querétaro, Querétaro 76240, Mexico; liturralde28@alumnos.uaq.mx; 3 Department of Mechanical Engineering, Universidad Tecnológica del Perú, Lima 15046, Peru; 4 Faculty of Engineering, Airport Campus, Autonomous University of Querétaro, Querétaro 76010, Mexico; rufino@uaq.mx

**Keywords:** nanostructured semiconductor photocatalysts, photocatalytic reactors, sacrificial agents, semiconductors, photocatalysis, hydrogen evolution, water splitting

## Abstract

Semiconductor-based photocatalytic water splitting is a promising pathway for sustainable hydrogen production; however, the reported performance depends not only on the intrinsic properties of the photocatalyst but also on reactor configuration and operating conditions. This systematic technical review examines the interplay between nanostructured semiconductor photocatalysts and the principal engineering variables governing photocatalytic hydrogen evolution. Particular attention is given to particle size, morphology, surface area, defect density, heterojunction design, cocatalyst incorporation, aggregation, and catalyst immobilization, as well as their interaction with reactor geometry, optical path length, photon distribution, catalyst loading, working volume, pH, sacrificial agents, mixing, thermal control, gas purging, and product quantification. The reviewed evidence indicates that these material and reactor parameters jointly determine light absorption, charge-carrier separation and transfer, suspension turbidity, mass transport, catalyst recovery, stability, and the measured hydrogen evolution rate. Batch slurry reactors remain the most widely used laboratory configuration, whereas annular, flat-panel, microreactor, fixed-bed, continuous-flow, and photofluidized systems offer specific advantages for photon utilization, catalyst reuse, product removal, and scale-up. The review also emphasizes the need to distinguish overall water splitting from sacrificial-agent-assisted hydrogen evolution. Standardized reporting of photocatalyst properties, irradiance, spectral distribution, illuminated area, reactor dimensions, reaction atmosphere, and gas-analysis procedures is essential to improve reproducibility and enable reliable comparisons among nanostructured photocatalytic systems.

## 1. Introduction

The growing global energy demand and the persistent dependence on fossil fuels have intensified environmental problems, including greenhouse gas emissions, climate change, and atmospheric pollution. In this context, the search for sustainable technologies capable of producing clean energy has become a strategic priority at both scientific and industrial levels. Among the emerging alternatives, hydrogen has been recognized as a promising energy carrier due to its high energy density and its combustion, which generates only water as a byproduct. However, most of the hydrogen currently produced originates from fossil-fuel-based processes, which limits its environmental sustainability. For this reason, photocatalytic hydrogen production via water splitting with semiconductor materials has attracted increasing attention as a potential route for the direct utilization of solar energy to generate clean fuels  [1,2,3].

Despite the significant advances achieved in the development of photocatalytic materials, several limitations related to experimental reproducibility, low overall conversion efficiency, and lack of standardization in operating conditions still persist. Most studies have primarily focused on modifying the electronic and structural properties of semiconductors, while fundamental variables such as reactor configuration, irradiation distribution, optical geometry, thermal control, agitation, pH, and inert gas purging are often treated as secondary parameters or reported only briefly. This situation hinders objective comparison across studies and limits a comprehensive understanding of the factors that govern photocatalytic hydrogen production [4,5,6].

The object of study in this research is the reactor configurations and experimental conditions employed in photocatalytic hydrogen production systems based on semiconductor materials. The operation of these systems relies on the absorption of radiation with sufficient energy to promote electrons from the valence band to the conduction band of the semiconductor, generating electron–hole pairs capable of driving the hydrogen evolution and oxygen evolution reactions. In recent years, research in this field has evolved from conventional batch configurations toward more advanced systems, including annular reactors, microreactors, continuous-flow reactors, and photofluidized configurations aimed at improving photon utilization and process scalability. Likewise, the integration of solar simulators, LED systems, and advanced irradiation distribution strategies reflects the current interest in developing more efficient technologies closer to real industrial applications [7,8,9].

Within the analyzed photocatalytic systems, both quantitative and qualitative variables directly influence hydrogen production. Among the quantitative variables are irradiation intensity, working volume, photocatalyst concentration, lamp power, purging time, stirring speed, pH, and hydrogen evolution rate. On the other hand, qualitative variables include reactor geometric configuration, semiconductor type, sacrificial agent type, irradiation source type, thermal control strategy, and system operation mode. The simultaneous interaction among these variables determines photocatalytic efficiency, photon distribution, mass transfer, and the experimental reproducibility reported in the scientific literature [10,11].

Based on this problem, several research questions arise in order to comprehensively understand the behavior of photocatalytic hydrogen production systems. The main research question is as follows: What reactor configurations are most commonly used for photocatalytic hydrogen production with semiconductor materials? Which experimental operating conditions most significantly affect process reproducibility and efficiency? How do variables such as pH, irradiation, photocatalyst concentration, sacrificial agents, and reactor geometry influence hydrogen evolution? What methodological limitations currently hinder the comparison and standardization of results among different studies?

Therefore, the objective of this research is to systematically analyze the reactor configurations and main experimental conditions employed in semiconductor-based photocatalytic hydrogen production systems, with the aim of identifying operational trends, critical variables, methodological limitations, and opportunities for improvement to increase the efficiency, reproducibility, and scalability of photocatalytic water-splitting systems.

## 2. Materials and Methods

This study was developed as a systematic literature review with a technical-analytical approach, aimed at identifying, organizing, and critically evaluating the current scientific evidence on reactor configurations and experimental operating conditions for photocatalytic hydrogen production from water using semiconductor materials. The methodological design was structured to provide a reproducible and transparent framework for the identification, screening, selection, and analysis of relevant studies, while also enabling the interpretation of technological and experimental trends reported in the field.

Given the multidisciplinary nature of the topic, which integrates photocatalysis, semiconductor materials, reactor engineering, light-driven processes, and experimental optimization, the review combined a systematic search strategy with a narrative and comparative synthesis. This approach was considered appropriate because it allowed not only the retrieval of relevant scientific publications but also the structured interpretation of the methodological heterogeneity commonly observed in photocatalytic hydrogen evolution studies.

To ensure methodological transparency and coherence, the review process was inspired by the principles of the PRISMA (Preferred Reporting Items for Systematic Reviews and Meta-Analyses) framework. Although this work does not constitute a clinical systematic review, the PRISMA logic was adopted as an organizational and reporting guideline to structure the stages of identification, screening, eligibility, and final inclusion of the literature corpus. In this study, PRISMA was not used merely as a formal reporting tool but as a methodological scaffold to define the progression from a broad initial search toward a more refined and thematically focused body of literature.

The overall methodological process was organized into four sequential stages: (i) initial literature identification, (ii) search refinement and temporal delimitation, (iii) eligibility assessment through thematic and technical criteria, and (iv) analytical classification of the selected studies according to reactor configuration and experimental variables.

This systematic review was retrospectively registered in the Open Science Framework (OSF) registry under the following reference: [12]. The registration of the review protocol ensures transparency, provides public access to the methodological design, and supports the reproducibility of the study.

### 2.1. Type of Study and Review Design

This work is classified as a systematic literature review complemented by a narrative-analytical synthesis. The systematic component enabled the definition of explicit search equations, temporal filters, and exclusion criteria, while the narrative component facilitated the interpretation of the selected studies in terms of experimental trends, methodological consistency, and reactor-related performance factors.

This design was considered particularly suitable for the present topic because photocatalytic hydrogen evolution studies frequently exhibit substantial variation in reactor geometry, catalyst loading, sacrificial media, pH, irradiation conditions, and gas handling procedures. Therefore, beyond identifying the existing literature, the review sought to compare how these operational variables are implemented and reported across the field.

The review was guided by the following research questions:

- RQ1: What reactor configurations are most frequently used for photocatalytic H_2_ production from water using semiconductor materials?
- RQ2: Which experimental operating conditions are most commonly reported in photocatalytic hydrogen evolution studies?
- RQ3: How do variables such as catalyst loading, pH, irradiation source, sacrificial agents, agitation, and inert gas purging influence the reproducibility and interpretation of H_2_ production results?
- RQ4: What methodological limitations currently hinder the standardization and comparability of photocatalytic hydrogen production experiments?

### 2.2. Information Sources and Search Strategy

The literature search was conducted primarily in the Scopus database, selected for its broad coverage of peer-reviewed scientific publications in the fields of photocatalysis, hydrogen production, semiconductor materials, materials science, and chemical engineering. Scopus was considered particularly suitable for this review because it allows the use of structured Boolean search equations and advanced filtering tools that support a reproducible and traceable search strategy.

The selection of Scopus as the primary source for the systematic corpus was a methodological decision intended to preserve the uniformity, traceability, and reproducibility of the search process. This database provides broad coverage of peer-reviewed international journals in photocatalysis, materials science, chemistry, and reactor engineering, including numerous publications that are also indexed in other recognized platforms. Its indexing requirements and filtering tools also helped to limit the inclusion of sources with insufficient editorial transparency or questionable reliability. Scopus was used to construct the systematic and bibliometric corpus; nevertheless, seminal publications and other relevant documents were identified through reference-list examination and citation tracking and were incorporated into the theoretical background and narrative discussion when appropriate.

The search strategy was designed to balance thematic specificity and retrieval flexibility. In contrast to highly restrictive searches that may omit relevant reactor or operational studies, the present strategy incorporated a controlled combination of terms associated with four major conceptual axes:

1. **Photocatalytic hydrogen production and water splitting;**
2. **Semiconductor-based photocatalysts;**
3. **Reactor configuration and photoreactor design;**
4. **Experimental operating conditions.**

The main search equation used in Scopus was the following:



TITLE-ABS-KEY(
(“photocatalytic hydrogen production” OR “photocatalytic hydrogen evolution”
OR “photocatalytic water splitting” OR “water splitting” OR “hydrogen evolution”)
AND
(semiconductor* OR photocatalyst* OR “semiconductor material*”)
AND
(reactor* OR photoreactor OR “reactor configuration” OR “reactor design”
OR “reaction system” OR “experimental condition*” OR pH OR irradiation
OR “light source” OR “sacrificial agent*” OR “catalyst loading”
OR stirring OR agitation OR temperature OR purging OR “inert gas”)
)
AND PUBYEAR > 2019 AND PUBYEAR < 2027
AND (EXCLUDE(SRCTYPE,“d”) OR EXCLUDE(SRCTYPE,“p”))
AND (EXCLUDE(DOCTYPE,“no”) OR EXCLUDE(DOCTYPE,“sh”) OR EXCLUDE(DOCTYPE,“ed”)
OR EXCLUDE(DOCTYPE,“dp”) OR EXCLUDE(DOCTYPE,“bk”) OR EXCLUDE(DOCTYPE,“tb”)
OR EXCLUDE(DOCTYPE,“cr”) OR EXCLUDE(DOCTYPE,“ch”) OR EXCLUDE(DOCTYPE,“cp”))
AND (EXCLUDE(SUBJAREA,“NURS”) OR EXCLUDE(SUBJAREA,“MEDI”)
OR EXCLUDE(SUBJAREA,“PSYC”) OR EXCLUDE(SUBJAREA,“ARTS”)
OR EXCLUDE(SUBJAREA,“SOCI”) OR EXCLUDE(SUBJAREA,“BUSI”)
OR EXCLUDE(SUBJAREA,“ECON”) OR EXCLUDE(SUBJAREA,“PHYS”)
OR EXCLUDE(SUBJAREA,“ENVI”) OR EXCLUDE(SUBJAREA,“BIOC”)
OR EXCLUDE(SUBJAREA,“AGRI”) OR EXCLUDE(SUBJAREA,“PHAR”)
OR EXCLUDE(SUBJAREA,“DENT”) OR EXCLUDE(SUBJAREA,“EART”)
OR EXCLUDE(SUBJAREA,“COMP”) OR EXCLUDE(SUBJAREA,“MATH”)
OR EXCLUDE(SUBJAREA,“CHEM”) OR EXCLUDE(SUBJAREA,“CENG”))
AND (EXCLUDE(EXACTKEYWORD,“Fuel Cell”) OR EXCLUDE(EXACTKEYWORD,“Battery”)
OR EXCLUDE(EXACTKEYWORD,“Supercapacitor”)
OR EXCLUDE(EXACTKEYWORD,“Wastewater Treatment”)
OR EXCLUDE(EXACTKEYWORD,“Dye Degradation”)
OR EXCLUDE(EXACTKEYWORD,“Pollutant Removal”)
OR EXCLUDE(EXACTKEYWORD,“Light”)
OR EXCLUDE(EXACTKEYWORD,“Charge Transfer”)
OR EXCLUDE(EXACTKEYWORD,“Carbon”)
OR EXCLUDE(EXACTKEYWORD,“Morphology”)
OR EXCLUDE(EXACTKEYWORD,“Bismuth Compounds”)
OR EXCLUDE(EXACTKEYWORD,“Water Splitting”)
OR EXCLUDE(EXACTKEYWORD,“Heterojunctions”)
OR EXCLUDE(EXACTKEYWORD,“Titanium Dioxide”)
OR EXCLUDE(EXACTKEYWORD,“Article”)
OR EXCLUDE(EXACTKEYWORD,“]+ Catalyst”)
OR EXCLUDE(EXACTKEYWORD,“Laser Beams”))



The search was limited to the period 2020–2026, with the intention of capturing recent developments in photocatalytic reactor design, semiconductor-based hydrogen production, and experimental optimization. Additional exclusion filters were applied to reduce thematic dispersion and remove document types, source types, and keywords considered peripheral to the specific scope of the review.

### 2.3. Inclusion and Exclusion Criteria

To ensure the thematic consistency and technical relevance of the selected literature, explicit inclusion and exclusion criteria were established prior to the screening process.

#### 2.3.1. Inclusion Criteria

Studies were included if they met one or more of the following criteria:

- Published between 2020 and 2026.
- Focused on photocatalytic hydrogen production, photocatalytic hydrogen evolution, or semiconductor-based water splitting.
- Included information related to reactor configuration, photoreactor design, or experimental operating conditions.
- Reported one or more variables such as catalyst loading, working volume, pH, irradiation source, sacrificial agents, temperature, stirring, or inert gas purging.
- Used semiconductor-based photocatalysts, including oxides, sulfides, nitrides, oxynitrides, carbon nitride-based systems, or hybrid semiconductor structures.
- Corresponded to peer-reviewed journal articles or relevant review papers with sufficient methodological detail.

#### 2.3.2. Exclusion Criteria

Studies were excluded if they met one or more of the following conditions:

- Published before 2020.
- Focused exclusively on electrocatalysis, photoelectrochemical cells, fuel cells, battery systems, or supercapacitors without direct relation to suspended or heterogeneous photocatalytic H_2_ evolution.
- Addressed pollutant degradation, wastewater treatment, CO_2_ reduction, or unrelated photocatalytic applications without specific relevance to hydrogen production.
- Presented only material synthesis or characterization results without meaningful discussion of hydrogen evolution performance or experimental conditions.
- Corresponded to editorials, notes, book chapters, conference papers, or other document types excluded in the search strategy.
- Lacked sufficient experimental or methodological detail for comparative analysis.

### 2.4. Selection and Eligibility Process

**Figure 1:**
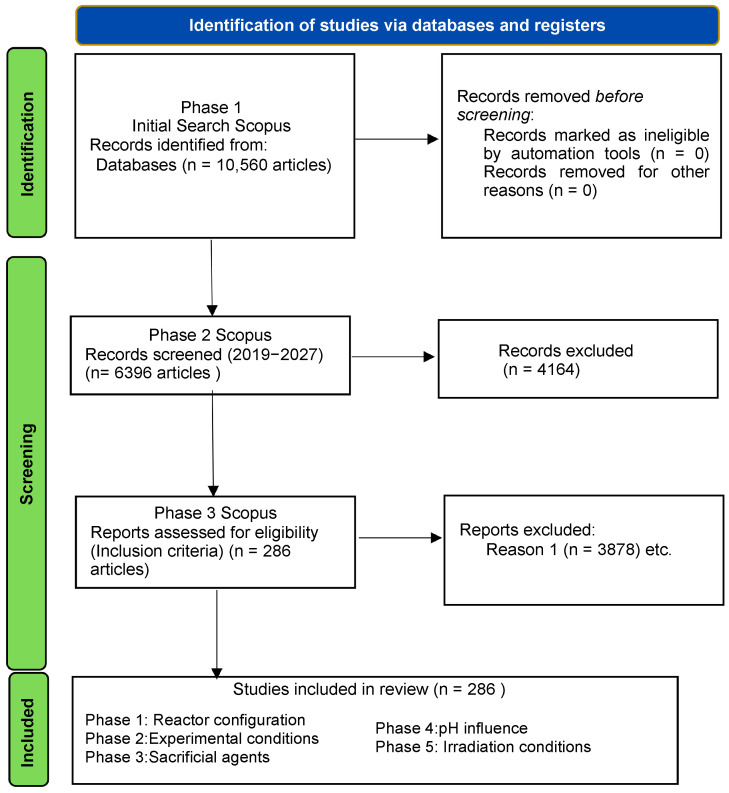
PRISMA-inspired flowchart of the literature identification, screening, and eligibility process used in this review on reactor configurations and experimental conditions for photocatalytic H_2_ production.

The identification, screening, and eligibility process followed a PRISMA-inspired progression, adapted to the scope of an engineering and materials-oriented review. The logic of this process is summarized schematically in Figure 1, where the review pathway is represented through sequential phases of literature reduction and thematic refinement.

As shown in the methodological flowchart, the review process began with an initial search in Scopus, from which 10,560 records were retrieved. This first stage corresponds to the identification phase, in which a broad query was intentionally used to maximize the retrieval of potentially relevant studies associated with photocatalytic hydrogen production and reactor-related terminology.

In the second stage, corresponding to the screening phase, the search was refined by applying the predefined temporal window and thematic restrictions. This refinement reduced the dataset to 6396 records, thereby eliminating studies outside the target period or outside the conceptual boundaries of the review.

The third stage corresponds to the eligibility phase, in which titles, abstracts, keywords, and methodological relevance were examined in greater detail. At this stage, studies that did not provide sufficient information regarding reactor configuration, semiconductor photocatalysts, or experimental operating conditions were excluded. As a result, the literature corpus was reduced to 286 eligible studies, which constituted the final analytical basis of the review.

This staged reduction reflects the application of PRISMA principles to an engineering review context, where the objective is not only to narrow the literature quantitatively but also to improve the thematic coherence and analytical usefulness of the final study set.

The exclusion decisions applied during screening and eligibility assessment were based on the search filters described in Section 2.2 and the explicit inclusion and exclusion criteria presented in Section 2.3. In particular, the publication period, document type, thematic relevance, direct relationship with photocatalytic hydrogen production, and availability of information on reactor configuration or experimental conditions were verified. Screening was performed through the examination of titles, abstracts, keywords, and methodological relevance. No automated tools were used to determine the inclusion or exclusion of the documents. Figure 1 provides a concise representation of the general identification, screening, eligibility, and inclusion flow, whereas the detailed explanation of the criteria is provided in the methodological sections indicated above.

### 2.5. Bibliometric Analysis and Thematic Mapping

As a complement to the systematic review process, a bibliometric co-occurrence analysis of keywords was performed using the VOSviewer 1.6.21 software. This analysis was intended to identify the dominant conceptual structures, thematic relationships, and emerging lines of research within the literature corpus associated with photocatalytic hydrogen production using semiconductor materials.

The resulting network map revealed that the field is strongly centered around terms such as hydrogen production, photocatalytic activity, photocatalytic water splitting, hydrogen evolution reaction, and photocatalytic hydrogen production. At the same time, the network shows strong associations with terms related to semiconductor engineering, co-catalysts, sacrificial agents, irradiation, oxygen vacancies, graphitic carbon nitride, layered semiconductors, and reaction kinetics, indicating that the literature is not only focused on hydrogen evolution performance but also on the material and mechanistic factors that govern it.

In thematic terms, the bibliometric network suggests the presence of several interconnected clusters, which may be broadly interpreted as follows:

1. Photocatalytic hydrogen evolution and water splitting performance
2. Semiconductor material design and defect engineering
3. Layered and two-dimensional photocatalytic materials
4. Surface chemistry, co-catalysts, and reaction kinetics
5. Irradiation conditions and energy conversion efficiency

This bibliometric structure supports the methodological organization adopted in the present review, confirming that reactor configuration and operating conditions must be analyzed in close connection with photocatalyst composition, surface engineering, and light-driven performance descriptors.

**Figure 2:**
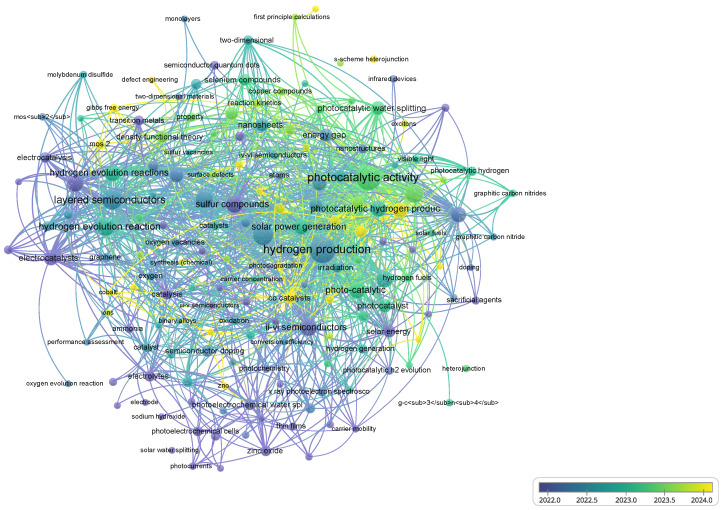
Keyword co-occurrence map generated with VOSviewer, showing the main conceptual relationships in the literature on semiconductor-based photocatalytic hydrogen production.

The keyword co-occurrence map generated from the analyzed literature corpus is shown in Figure 2, where the main conceptual relationships among hydrogen production, photocatalytic activity, semiconductor materials, irradiation conditions, co-catalysts, and related mechanistic descriptors can be observed.

### 2.6. Data Extraction and Analytical Approach

Once the final set of eligible studies was defined, a structured data extraction process was carried out to collect the most relevant technical and methodological information from each publication.

For each selected article, variables such as the following were systematically recorded:

- Publication year;
- Authors and journal source;
- Semiconductor material or photocatalyst type;
- Reactor configuration and reactor material;
- Working volume;
- Catalyst loading;
- Type and concentration of sacrificial agent;
- pH conditions;
- Irradiation source and spectral characteristics;
- Irradiation intensity or lamp power (when reported);
- Stirring or mixing conditions;
- Temperature control strategy;
- Inert gas purging conditions;
- H_2_ evolution rate and reported efficiency metrics;
- Methodological limitations or reproducibility issues.

Subsequently, the selected studies were organized and analyzed according to four major comparative dimensions:

1. **Reactor configuration**: batch reactors, annular reactors, flat-panel systems, microreactors, fixed-bed systems, and continuous-flow configurations.
2. **Experimental operating conditions**: catalyst loading, working volume, pH, sacrificial media, irradiation, stirring, temperature, and purging.
3. **Photocatalyst characteristics**: semiconductor family, bandgap-related properties, co-catalyst incorporation, and structural features.
4. **Performance descriptors**: H_2_ production rate, apparent quantum efficiency, and other experimental response indicators.

Finally, the analysis of the literature was conducted under a **qualitative-comparative and technically interpretive approach**, aimed at identifying recurrent methodological patterns, experimental inconsistencies, common reporting gaps, and opportunities for improving reproducibility and standardization in photocatalytic hydrogen production studies.

### 2.7. Preliminary Discussion of the Initial Search

The initial search stage provided a broad and useful overview of the scientific landscape surrounding semiconductor-based photocatalytic hydrogen production. Although this first retrieval was intentionally expansive, its main value did not lie solely in the number of records identified, but rather in the possibility of recognizing the dominant conceptual directions, thematic overlaps, and methodological dispersion that characterize the field. In this sense, the initial search functioned not only as a record identification step but also as a diagnostic phase for understanding the structure of the available literature prior to thematic refinement.

From the first Scopus query, a total of 10,560 records were retrieved. This high number confirms that the broader field of hydrogen-related photocatalysis is currently a highly active research domain. However, the volume of retrieved publications also revealed an important methodological challenge: the scientific literature associated with photocatalytic hydrogen production is deeply interconnected with adjacent topics such as material synthesis, defect engineering, surface chemistry, photoelectrochemistry, pollutant degradation, and general photocatalytic activity. Consequently, the first search did not exclusively capture literature focused on reactor configuration and experimental operation but rather a much wider research ecosystem in which reactor-oriented studies represent only one subset [13,14].

This observation is particularly relevant because it confirms that the terminology used in photocatalytic hydrogen research is often material-centered rather than reactor-centered. In many cases, studies are indexed under terms related to semiconductor composition, bandgap engineering, heterojunction design, co-catalyst loading, or charge separation mechanisms, even when they also contain valuable experimental information regarding reactor setup or operational conditions. Therefore, an excessively restrictive search focused only on explicit reactor-related terminology could have excluded a substantial number of experimentally useful studies.

#### 2.7.1. General Thematic Dispersion in the Initial Search

A first inspection of the retrieved records revealed a broad thematic dispersion across several recurring lines of research. While all of these areas are connected to hydrogen evolution or photocatalysis, not all of them are equally relevant to the specific objective of this review. In particular, the initial search captured a significant number of studies centered primarily on material development and performance enhancement, often with limited attention to the experimental architecture of the reaction system.

This trend suggests that, in the current literature, photocatalytic hydrogen production is still predominantly approached from a materials science perspective, whereas reactor engineering and methodological standardization are comparatively less consolidated research dimensions. Such imbalance is methodologically important, since the performance of a photocatalyst is often interpreted mainly through intrinsic material properties, while the influence of the experimental environment is underreported or insufficiently discussed.

To clarify this thematic dispersion, the main domains identified during the initial literature search, their typical focus, and their relevance to the scope of this review are summarized in Table 1.

The preliminary search, therefore, revealed that the field contains a large amount of potentially relevant literature but also a considerable amount of thematic overlap that must be carefully filtered. This justifies the use of a staged screening and eligibility process rather than relying exclusively on keyword restriction at the search equation level.

#### 2.7.2. Interpretation of the Keyword Co-Occurrence Structure

The bibliometric mapping performed through VOSviewer further supported the interpretation of the initial search as a broad thematic field with clustered internal specialization. As observed in the co-occurrence network, the most central and highly connected terms are hydrogen production, photocatalytic activity, photocatalytic water splitting, hydrogen evolution reaction, and photocatalyst, indicating that the literature is strongly organized around hydrogen generation performance and photocatalyst functionality.

At the same time, the map also reveals the strong presence of related terms such as graphitic carbon nitride, layered semiconductors, oxygen vacancies, co-catalysts, sacrificial agents, irradiation, reaction kinetics, and photoelectrochemical water splitting, suggesting that the literature is fragmented into multiple but interconnected subdomains. This distribution confirms that hydrogen evolution is not studied in isolation but rather as part of a broader network of material, mechanistic, and energy-conversion research themes.

A particularly important observation from the keyword network is that terms directly associated with reactor design, hydrodynamics, working volume, purging strategy, or thermal control do not dominate the visible core of the field to the same extent as material or photocatalyst descriptors. This indicates that reactor-oriented knowledge is present in the literature, but often embedded within broader photocatalytic studies rather than treated as a central standalone topic. As a result, extracting reactor and operational insights requires a more interpretive and comparative reading of the literature rather than a purely keyword-based filtering strategy.

Overall, the keyword structure confirms that the field is highly interconnected and that reactor-related experimental knowledge is often dispersed across studies primarily focused on material performance or mechanistic enhancement. This observation strongly supports the need for a review specifically dedicated to organizing and comparing reactor configurations and experimental conditions, rather than only photocatalyst composition.

To further clarify the structure of the keyword co-occurrence network, the main observed keyword groups, their representative terms, preliminary interpretation, and relevance to the scope of this review are summarized in Table 2.

#### 2.7.3. Methodological Need for Search Refinement

One of the main insights obtained from the preliminary search was the methodological necessity of refining the literature corpus before entering the detailed review stage. Although the first search was successful in capturing the breadth of the field, its inclusiveness also introduced a substantial number of studies that were only tangentially relevant to the central objective of the present review.

In practical terms, three major types of literature were found to contribute to this initial overexpansion:

1. **Material-centric studies** focused almost exclusively on synthesis, morphology, and band structure engineering, with limited reporting of experimental operating conditions.
2. **Adjacent hydrogen-related technologies**, including photoelectrochemical and electrocatalytic systems, which often use similar terminology but involve substantially different experimental architectures.
3. **General photocatalytic applications**, especially pollutant degradation and environmental remediation studies, where hydrogen evolution is not the primary performance target.

This confirms that the main challenge at the initial search stage was not a lack of available literature, but rather the need to discriminate between thematic proximity and actual analytical relevance. Consequently, the refinement process was not merely a reduction in the number of documents but a necessary methodological step to transform a broad scientific domain into a coherent and reviewable corpus.

To make this refinement criterion explicit, the main sources of thematic noise identified during the initial search, together with their origin, relevance to the review scope, and filtering rationale, are summarized in Table 3.

This preliminary filtering logic is particularly important from a review design perspective because it justifies why the final corpus was not selected simply by quantitative reduction but by technical relevance to reactor configuration and operating conditions. In other words, the transition from the initial search to the eligible dataset reflects a conceptual refinement rather than a merely numerical screening exercise.

#### 2.7.4. Preliminary Discussion and Relevance to the Present Review

From a broader analytical perspective, the initial search confirmed two key points that support the relevance of the present article. First, the scientific literature on photocatalytic hydrogen production is abundant, rapidly expanding, and strongly diversified. Second, despite this large body of work, the specific relationship between reactor configuration, operational parameters, and hydrogen evolution performance remains fragmented across studies and is rarely addressed as the central object of analysis.

This fragmentation creates an important gap in the field. While many publications report hydrogen evolution rates and photocatalyst performance enhancements, fewer studies systematically compare the experimental architectures under which those results were obtained. As a consequence, the literature often presents catalyst-centered conclusions without sufficiently clarifying the extent to which the observed performance depends on the surrounding reaction environment.

Therefore, the preliminary analysis of the initial search not only justified the need for further filtering but also reinforced the scientific motivation of this review: to reorganize a dispersed body of knowledge into a structured comparative framework centered on reactor design, experimental conditions, and their implications for H_2_ production reproducibility and interpretation.

## 3. Theoretical Foundations

Semiconductor-mediated water splitting is fundamentally based on the generation of electron–hole pairs upon absorption of photons with energy equal to or greater than the bandgap of the material. The pioneering work of Fujishima and Honda demonstrated that an illuminated semiconductor electrode, specifically TiO_2_, can promote spatial charge separation and drive interfacial redox reactions, thereby establishing the photo(electro)chemical paradigm for water decomposition [15].

**Figure 3:**
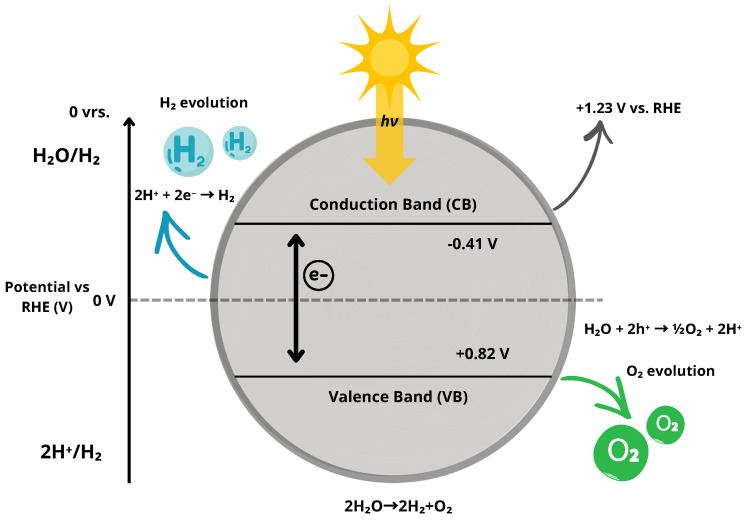
Thermodynamic potential required for overall water splitting under standard conditions (25 °C, 1 atm) referenced to the reversible hydrogen electrode (RHE). The hydrogen evolution reaction (HER) occurs at 0 V vs. RHE, while the oxygen evolution reaction (OER) takes place at +1.23 V vs. RHE. In practical systems, additional voltage is required to overcome kinetic overpotentials and resistive losses.

From a thermodynamic perspective, for the overall water-splitting reaction to proceed, the band-edge positions of the semiconductor must appropriately straddle the reduction and oxidation potentials of the *H*^+^/*H*_2_ and *H*_2_*O*/*O*_2_ redox couples, respectively, while considering the solution pH and the electrochemical reference scale used (Figure 3). In this context, material development efforts have focused on designing photocatalysts with suitable band alignment, chemical stability, efficient optical absorption, and favorable surface reaction kinetics. In addition, the incorporation of cocatalysts is commonly employed to reduce kinetic overpotentials, facilitate charge transfer, and suppress electron–hole recombination pathways [16,17].

Photocatalytic hydrogen production from water is therefore a multivariable process governed by the strong coupling between semiconductor physics, interfacial electrochemistry, charge carrier dynamics, light harvesting, and reactor engineering. Although the development of advanced photocatalysts has received substantial attention in recent years, the overall efficiency of hydrogen evolution is not determined solely by the intrinsic properties of the semiconductor. Rather, it results from the combined influence of band structure, carrier separation and transport, surface catalytic activity, reactor geometry, light distribution, and physicochemical operating conditions. Consequently, a rigorous theoretical understanding of photocatalytic water splitting requires an integrated analysis of these factors, particularly when comparing the performance of different photocatalytic systems under distinct experimental configurations and operating conditions [18,19].

### 3.1. Principles of Semiconductor-Based Water Photolysis

Semiconductor-based water photolysis relies on the ability of a photoactive material to absorb photons and convert the absorbed radiant energy into electron–hole pairs capable of driving redox reactions at the solid–liquid interface. When a semiconductor absorbs photons with energy equal to or greater than its bandgap energy ($E_{g}$), an electron is excited from the valence band (VB) to the conduction band (CB), leaving behind a hole in the VB. If these charge carriers are successfully separated and transferred to the catalyst surface before recombination occurs, they can participate in the hydrogen evolution reaction (HER) and oxygen evolution reaction (OER) [18,20].

The overall water splitting reaction can be expressed as

(1) $$2H_{2}O\left(l\right)\to 2H_{2}\left(g\right)+O_{2}\left(g\right)$$

with a standard Gibbs free energy change of

(2) $$\Delta G{}^{\circ}=+237.2\;\mathrm{kJ}\;{\mathrm{mol}}^{-1}$$

which corresponds to a minimum thermodynamic potential of

(3) ΔE°=1.23V

under standard conditions. However, this theoretical value represents only the ideal energy requirement. In practical systems, additional energetic losses arise due to kinetic overpotentials, charge recombination, interfacial resistance, and mass transport limitations, meaning that the effective energetic demand is generally higher than the thermodynamic minimum [19,20].

For a semiconductor to be suitable for photocatalytic water splitting, its band edges must satisfy specific energetic requirements. The bottom of the conduction band must be more negative than the reduction potential of $H^{+}/H_{2}$, while the top of the valence band must be more positive than the oxidation potential of $O_{2}/H_{2}O$:

(4) $$E_{CB}<E\left(H^{+}/H_{2}\right)$$

(5) $$E_{VB}>E\left(O_{2}/H_{2}O\right)$$

This condition ensures that photogenerated electrons possess sufficient reducing power to convert protons into hydrogen, while holes exhibit enough oxidizing ability to drive water oxidation. Consequently, not all photoactive semiconductors are intrinsically suitable for overall water splitting, even if they are able to produce hydrogen in the presence of sacrificial reagents [18,19].

Although band-edge alignment defines the thermodynamic feasibility of HER and OER, the overall photocatalytic response also depends on additional physicochemical and kinetic factors. Therefore, optical absorption, charge separation, chemical stability, and surface reaction kinetics must be considered together with the conduction- and valence-band positions, as summarized in Table 4.

A critical distinction must be made between *photocatalytic hydrogen evolution in the presence of sacrificial agents* and *overall water splitting*. In the former, compounds such as methanol, ethanol, triethanolamine (TEOA), or lactic acid act as hole scavengers, facilitating charge separation and significantly increasing hydrogen production rates. However, these systems do not represent complete water splitting, since the oxidation half-reaction is chemically assisted. Therefore, the comparison of hydrogen production results obtained with and without sacrificial agents must be performed with caution [18,21].

**Table 4:** Fundamental requirements for semiconductor-based water splitting.

Criterion	Theoretical Requirement	Practical Implication	Ref.
Optical absorption	$h\nu \geq E_{g}$	Enables photon absorption and the generation of photogenerated electron–hole pairs.	[18,19]
Conduction-band position	$E_{\mathrm{CB}}$ must be more negative than the $H^{+}/H_{2}$ reduction potential	Provides photogenerated electrons with sufficient reduction potential to drive the hydrogen evolution reaction (HER).	[20]
Valence-band position	$E_{\mathrm{VB}}$ must be more positive than the $O_{2}/H_{2}O$ oxidation potential	Provides photogenerated holes with sufficient oxidation potential to drive the oxygen evolution reaction (OER).	[20]
Charge separation	Reduced recombination and sufficiently long-lived photogenerated charge carriers	Charge separation typically occurs on ps–ns timescales, whereas recombination may occur from ps to μs. These processes may be evaluated through carrier-lifetime measurements, time-resolved photoluminescence, transient absorption spectroscopy, photocurrent measurements, or charge-transfer resistance. Effective charge separation increases the availability of electrons and holes for surface redox reactions.	[22]
Chemical stability	Resistance to photocorrosion under clearly defined reaction conditions	May be evaluated through photocatalytic activity retention after a specified irradiation time or number of cycles; the concentration of leached species; and the preservation of the crystalline phase, chemical composition, and surface properties after reaction. These indicators support the assessment of long-term photocatalytic stability.	[19]
Surface kinetics	Availability of active sites and sufficiently low kinetic barriers for HER and OER	Controls interfacial charge transfer, the adsorption and transformation of reaction intermediates, and the formation of molecular H_2_ and O_2_.	[18]

From a mechanistic perspective, photocatalytic water splitting can be interpreted as a competition between two opposite pathways: a productive route in which absorbed photon energy is converted into chemical bonds, and a dissipative route dominated by charge recombination, thermal relaxation, and parasitic surface reactions. This explains why a semiconductor with an adequate bandgap does not necessarily exhibit high photocatalytic activity. In practice, hydrogen evolution depends not only on the thermodynamic feasibility of the process but also on the efficiency with which the absorbed energy is transformed into long-lived, chemically useful charge carriers [18,19].

### 3.2. Generation and Separation of Photoinduced Charges

Following photoexcitation, the efficiency of photocatalytic hydrogen production is largely governed by the fate of the photogenerated charge carriers. When a photon with sufficient energy is absorbed, an electron is promoted from the valence band to the conduction band, leaving a hole in the valence band. These electrons and holes must then be efficiently separated and transported toward surface active sites, where they participate in the hydrogen and oxygen evolution half-reactions. However, charge recombination can occur either in the bulk or at the surface, often on ultrafast timescales ranging from femtoseconds to nanoseconds. If recombination takes place before interfacial charge transfer, the absorbed photon energy is dissipated as heat or radiative emission rather than being converted into chemical energy [18,22].

This limitation is particularly relevant in conventional semiconductors such as TiO_2_, where rapid recombination strongly restricts photocatalytic efficiency. Therefore, strategies such as defect engineering, heterojunction design, morphology control, and the incorporation of cocatalysts have become essential to promote charge separation, extend carrier lifetimes, facilitate charge extraction, and improve the kinetics of the hydrogen and oxygen evolution reactions [16,23].

From a broader perspective, photocatalysis can be understood as a coupled sequence of processes that must be simultaneously optimized: optical absorption, charge separation and transport, interfacial charge transfer, and surface catalytic kinetics. Recent “photocatalysis-by-design” approaches emphasize that the rational improvement of photocatalytic systems requires not only the design of materials with favorable electronic and surface properties but also the quantitative evaluation of each step under well-defined and normalized experimental conditions. Without proper control of these sequential processes and standardized reporting of reaction parameters, comparisons among different photocatalysts, reactor geometries, and irradiation conditions may become ambiguous or even misleading [24,25].

The global photocatalytic efficiency can be interpreted as the product of several partial efficiencies:

(6) $$\eta_{global}=\eta_{abs}\cdot \eta_{sep}\cdot \eta_{trans}\cdot \eta_{surf}$$

where $\eta_{abs}$ is the photon absorption efficiency, $\eta_{sep}$ is the charge separation efficiency, $\eta_{trans}$ is the charge transport efficiency, and $\eta_{surf}$ is the interfacial surface reaction efficiency. This expression illustrates that a photocatalyst with strong optical absorption may still perform poorly if it suffers from severe recombination or sluggish surface kinetics [18,19].

Several strategies have been proposed to improve charge separation, including the construction of heterojunctions, Z-scheme systems, S-scheme architectures, co-catalyst loading, and defect engineering. Co-catalysts such as Pt, Ni, Ru, MoS_2_, and transition metal sulfides can act as electron sinks, lowering the activation barrier for HER and promoting interfacial electron transfer. Nevertheless, the mere presence of a heterojunction or co-catalyst does not guarantee improved activity, as poor interfacial contact or unfavorable band alignment may instead introduce trap states that accelerate recombination [21,26,27].

A common misconception in the literature is to associate broader visible-light absorption directly with improved hydrogen production. In reality, optical absorption alone does not ensure photocatalytic performance if the photogenerated carriers are not efficiently separated and utilized. Thus, the relevant question is not only how much light is absorbed but what fraction of that energy is ultimately converted into usable electrons and holes for surface redox chemistry [18,19].

The main photoelectronic and interfacial processes involved in semiconductor photocatalysis, together with their representative timescales and impact on H_2_ production, are summarized in Table 5.

Because recombination, inefficient charge transport, and sluggish interfacial kinetics can limit the conversion of absorbed photons into chemical products, different material-design strategies have been proposed to improve charge separation and carrier utilization. These strategies include Type-II heterojunctions, Z-scheme and S-scheme architectures, co-catalyst loading, defect engineering, and nanostructuring. Their mechanisms, advantages, and main limitations are summarized in Table 6.

### 3.3. Influence of Reactor Geometry on Light Capture and Distribution

Photocatalytic hydrogen production is inherently governed by the interaction between light, matter, and the hydrodynamic environment of the reactor (Table 7). Therefore, the photoreactor should not be regarded merely as a vessel containing the photocatalyst suspension but as an optical and transport-controlled system that determines photon distribution, light penetration, catalyst exposure, mixing behavior, and gas evolution [28,29]. In heterogeneous photocatalysis, reactor geometry strongly influences the effective irradiation field within the reactive volume and, consequently, the apparent reaction rate. Parameters such as optical path length, illuminated area, wall reflection, light scattering, shading effects, beam confinement, illuminated fraction, and spatial intensity gradients can dominate the kinetic response, particularly when comparing slurry and immobilized systems [30,31].

In slurry-based systems, the propagation of radiation through the suspension is affected by absorption, scattering, particle shading, and attenuation. As a result, the effective irradiance experienced by the catalyst is not defined solely by the nominal lamp power but also by the catalyst concentration, reactor depth, illuminated surface, optical path length, and spatial arrangement of the light source. Excessive optical depth may generate poorly illuminated or even dark zones, where part of the suspended catalyst contributes only marginally to hydrogen production [28,32]. This aspect is particularly relevant because, under otherwise identical experimental conditions, the same catalytic formulation may exhibit markedly different hydrogen evolution rates depending on the optical regime imposed by the reactor design [33,34,35].

Several reactor configurations have been employed for photocatalytic hydrogen evolution, including conventional batch reactors, annular reactors, flat-panel systems, microreactors, fixed-bed arrangements, and continuous-flow designs. Each configuration involves specific trade-offs among photon utilization, mass transfer, gas disengagement, catalyst handling, and scalability. For example, annular and flat-panel reactors generally provide more uniform irradiation than conventional cylindrical vessels, while microreactors improve the surface-area-to-volume ratio and reduce optical depth, although often with limitations in processing capacity [28,29,32]. Consequently, when reporting photocatalytic performance, such as H_2_ evolution rate, apparent quantum yield (AQY), solar-to-hydrogen (STH) efficiency, or related metrics, it is essential to describe not only the photocatalyst composition but also the reactor geometry, irradiation mode, optical depth, illuminated area, flow or batch operation, and the methodology used to quantify the incident and/or absorbed photon flux. This level of detail is necessary to distinguish intrinsic photocatalytic activity from reactor-dependent effects and to enable meaningful comparison among different studies [30,31,33,34,35].

In this context, different reactor geometries offer specific advantages and limitations depending on the balance between photon utilization, mass transfer, catalyst handling, gas removal, and scalability. Therefore, the selection of reactor configuration can directly influence the apparent H_2_ production rate and the reproducibility of photocatalytic measurements. The main effects of representative reactor geometries on photocatalytic performance are summarized in Table 8.

An important methodological issue in the literature is that reactor design is often underreported or treated as secondary, even though it strongly affects the local photon field experienced by the catalyst. As a result, the apparent superiority of one photocatalyst over another may partially arise from differences in optical geometry rather than intrinsic material properties. This highlights the importance of systematically reporting reactor dimensions, lamp position, illuminated area, and optical path length when comparing photocatalytic hydrogen evolution data [28,29].

### 3.4. Effects of pH on the Thermodynamics and Kinetics of H_2_ Evolution

The pH of the reaction medium is one of the most influential variables in photocatalytic hydrogen production because it affects not only electrochemical thermodynamics but also surface charge, interfacial adsorption, semiconductor stability, proton/hydroxide availability, and proton-coupled electron transfer (PCET) mechanisms (Table 9 and Table 10). Therefore, pH should not be treated merely as an empirical operating parameter but as a variable that fundamentally reshapes the semiconductor–electrolyte interface [19,21].

From an electrochemical standpoint, the redox potentials of water-related half-reactions shift with pH according to the Nernst equation. In particular, the equilibrium potentials of the hydrogen and oxygen evolution reactions change by approximately 59 mV per pH unit at 25 °C. For the $H^{+}/H_{2}$ couple, this shift can be expressed as

(7) $$E(H^{+}/H_{2})=0-0.059\;\mathrm{pH}$$

This pH-dependent displacement modifies the apparent water stability window when potentials are referenced to the NHE/SHE scale, which explains the widespread use of the reversible hydrogen electrode (RHE) scale to normalize electrochemical potentials and facilitate comparisons under different pH conditions [36,37].

Beyond these thermodynamic effects, pH also plays a decisive role in photocatalytic kinetics. Variations in pH can modify the surface charge of the photocatalyst, particularly with respect to its point of zero charge, thereby influencing the adsorption of water molecules, protons, hydroxide ions, and other reactive intermediates. These changes directly affect interfacial charge transfer rates and the availability of species involved in hydrogen and oxygen evolution reactions. Consequently, even when the semiconductor exhibits suitable band-edge alignment for overall water splitting, the reaction may still be limited by sluggish surface kinetics, which are frequently improved through cocatalyst integration [17,38].

For this reason, meaningful comparisons of photocatalytic hydrogen production require precise reporting of the reaction pH, electrolyte or buffer composition, and reference scale used for electrochemical potentials. Without this information, differences in photocatalytic performance may reflect variations in interfacial chemistry and proton availability rather than intrinsic improvements in the semiconductor or cocatalyst system [19,21,38].

This means that the energetic alignment between the semiconductor band edges and the redox potentials of the medium depends on pH. In acidic media, proton reduction is thermodynamically more accessible, whereas in alkaline media, the lower concentration of free protons may alter the rate-determining steps of hydrogen evolution [20,21].

In addition to thermodynamics, pH also modifies the surface chemistry of the photocatalyst. Most oxide and sulfide semiconductors contain surface hydroxyl groups that can be protonated or deprotonated depending on the solution pH. As a result, the surface charge of the catalyst varies with respect to its point of zero charge (${\mathrm{pH}}_{PZC}$), influencing adsorption, electrostatic interactions, colloidal stability, and charge transfer at the interface [18,19].

The influence of pH is frequently reported in terms of an “optimal value”, but this interpretation can be misleading if it is not supported by mechanistic analysis. An increase in hydrogen evolution rate at a given pH does not necessarily indicate improved intrinsic catalytic performance; it may instead reflect changes in adsorption, band alignment, cocatalyst activation, or colloidal dispersion. Therefore, pH should be interpreted as a coupled electrochemical and interfacial variable rather than an isolated operating condition [19,21].

### 3.5. Relevance of Irradiation Spectrum and Power in Electronic Excitation

Light irradiation constitutes the fundamental energy input in photocatalytic hydrogen production; however, its effect on H_2_ evolution cannot be adequately described only by the nominal electrical power of the lamp (Table 11). The photoactive response of a semiconductor depends on the spectral overlap between the incident radiation and the optical absorption window of the photocatalyst, as well as on the irradiance reaching the sample, the spatial distribution of photons, and the uniformity of illumination [18,19]. In this regard, only photons with energies equal to or greater than the bandgap energy can promote band-to-band excitation, according to

(8) $$h\nu \geq E_{g}$$

where *h* is Planck’s constant, ν is the photon frequency, and $E_{g}$ is the bandgap energy of the semiconductor. Therefore, the irradiation spectrum (λ), together with the power density and photon flux, determines which fraction of the UV, visible, or near-infrared region can be effectively utilized by the photocatalyst. For this reason, quantitative studies and reviews recommend reporting irradiation conditions in terms of photon flux and spectral distribution rather than relying exclusively on lamp power (Table 12). Whenever possible, efficiency should also be assessed using metrics such as the apparent quantum yield (AQY) under monochromatic illumination, which enables a direct correlation between absorbed photons and H_2_ evolution, and the solar-to-hydrogen (STH) efficiency under simulated solar spectra, which provides a closer approximation to realistic operating conditions [39,40].

Furthermore, increases in light intensity do not necessarily lead to proportional improvements in hydrogen production. At high irradiance levels, the photocatalytic response may become limited by second-order electron–hole recombination, saturation of surface active sites, inefficient photon utilization, or mass-transfer constraints. As a result, the reactor configuration, optical path, catalyst loading, and illumination strategy become integral components of the measured photocatalytic performance, rather than merely external experimental parameters. Consequently, the literature emphasizes the need to normalize and clearly report how irradiance and spectral distribution are measured, including associated uncertainties, in order to avoid comparisons that conflate optical and reactor-related effects with the intrinsic properties of the photocatalyst [4,39].

Only photons with energies equal to or greater than the bandgap can generate electron–hole pairs. Therefore, the usefulness of a given light source depends on its ability to provide photons in the wavelength range that matches the bandgap of the photocatalyst. Wide-bandgap materials such as TiO_2_ and ZnO are mainly activated under UV light, whereas sulfides, oxynitrides, and carbon nitride-based systems may exploit a larger portion of the visible spectrum [22,27].

At low light intensities, the photocatalytic rate often increases approximately linearly with photon flux, since carrier generation is the limiting factor. However, at higher intensities, deviations from linearity may occur due to charge recombination, local heating, saturation of active sites, bubble coverage, or mass transfer limitations. Thus, increasing lamp power does not always lead to a proportional increase in hydrogen production [18,19].

A persistent limitation in the photocatalysis literature is that studies often report only the nominal lamp power (e.g., a 300 W Xe lamp) without specifying the actual irradiance at the sample, the spectral output, the illuminated area, or the distance between the source and the reactor. This lack of optical standardization makes inter-study comparisons difficult and may lead to misleading conclusions regarding catalyst performance. Scientifically, what matters is not merely how much electrical power the lamp consumes but how many useful photons effectively reach the catalyst and under what spatial and spectral conditions [19,28].

### 3.6. Integrated Perspective on Photocatalytic Hydrogen Production

The theoretical foundations of photocatalytic hydrogen production clearly demonstrate that system performance cannot be attributed to a single dominant factor. Instead, hydrogen evolution emerges from the coupled interaction of semiconductor band energetics, charge carrier dynamics, optical absorption, reactor geometry, interfacial chemistry, aqueous-medium conditions, and irradiation parameters. This multidimensional nature helps explain the broad dispersion of hydrogen evolution rates reported in the literature, even for photocatalysts that appear similar in composition, structure, or nominal operating conditions [18,19].

From a critical perspective, one of the main challenges in the field is not only the development of new photocatalytic materials but also the establishment of experimental and analytical criteria that enable rigorous comparison among studies. A semiconductor with favorable band positions may still exhibit poor performance if charge separation and transport are inefficient; a catalyst with broad visible-light absorption may underperform in an optically inefficient reactor; and an apparently optimal pH may reflect favorable interfacial reaction conditions rather than superior intrinsic activity. Therefore, any meaningful evaluation of photocatalytic hydrogen evolution must treat these variables as strongly coupled descriptors rather than independent parameters [21,28].

Overall, the efficiency of semiconductor-based water photolysis depends on a hierarchical sequence of events that includes photon absorption, generation of electron–hole pairs, charge separation and transport, interaction with the aqueous medium, and surface redox conversion under suitable optical, chemical, and geometric conditions. This integrated and quantitatively supported perspective is essential for the critical interpretation of photocatalytic performance and for establishing more reliable criteria for photocatalyst evaluation, reactor design, experimental standardization, and inter-study comparison [18,19,28].

To provide a quantitative connection between the theoretical principles discussed and the experimental performance reported in the literature, representative results from the reviewed studies are summarized here. For Pt-loaded g-C_3_N_4_, H_2_ evolution rates of approximately 114, 810, and 427 μmol g^−1^ h^−1^ were reported using ethanol, triethanolamine, and lactic acid, respectively, demonstrating that the oxidation medium and hole-scavenging efficiency can produce differences of more than sevenfold for a similar photocatalytic platform. CdS-based systems exhibited H_2_ evolution rates above 1000 μmol g^−1^ h^−1^ in methanol-containing media and above 1500 μmol g^−1^ h^−1^ in Na_2_S/Na_2_SO_3_ solutions, whereas TiO_2_ systems using formic acid generally produced approximately 300–500 μmol g^−1^ h^−1^. Likewise, the pH-dependent results indicate that some systems exhibit maximum activity under near-neutral conditions, while CdS/Pt commonly reaches values above 1000 μmol g^−1^ h^−1^ at approximately pH 8–9. These representative values, discussed in greater detail in Table 13, Table 14, Table 15, Table 16, Table 17, Table 18, Table 19, Table 20 and Table 21, quantitatively confirm that H_2_ evolution is strongly influenced by the coupling among semiconductor properties, charge separation, surface reactions, sacrificial-agent chemistry, and pH. The quantitative results presented in Table 13, Table 14, Table 15, Table 16, Table 17, Table 18, Table 19, Table 20, Table 21 and Table 22 should therefore be understood as representative performance ranges that illustrate the combined influence of the material, reactor, and experimental conditions.

## 4. Analysis of Results

### 4.1. Structural, Morphological, Optical, and Photocatalytic Characteristics of Representative Semiconductors

Although this review primarily focuses on reactor configuration and operating conditions, the intrinsic properties of semiconductor photocatalysts are also essential for interpreting H_2_ evolution. As summarized in Table 13, crystallinity, defect density, morphology, particle size, surface area, and bandgap directly affect light absorption, charge separation, carrier transport, and surface redox kinetics. However, these properties cannot be evaluated independently of irradiation conditions, catalyst loading, sacrificial agents, pH, and reactor geometry. Therefore, the reported H_2_ production should be interpreted as the combined result of material properties, reactor design, and operating conditions.

**Table 13:** Representative structural, morphological, optical, and photocatalytic characteristics of semiconductor-based H_2_ production systems.

Photocatalyst	Crystal Phase	Morphology	Size/Surface Area	$E_{g}$ (eV)	H_2_ Production	Experimental Conditions	Ref.
2.4 wt.% WS_2_–TiO_2_	Mixed-phase P25 TiO_2_ and layered WS_2_	Few-layer WS_2_ flakes coupled with TiO_2_ nanoparticles	TiO_2_: 20 nm; WS_2_: few-layer	WS_2_: 1.75; TiO_2_: 3.0	321 μmol g^−1^ h^−1^ ^a^	300 W Xe lamp (AM 1.5 filter); aqueous methanol/NaOH medium; optimized WS_2_ loading of 2.4 wt.%	[41]
TiO_2_/ZnIn_2_S_4_	Anatase TiO_2_ and hexagonal ZnIn_2_S_4_	1D/2D S-scheme heterointerface	BET area ≈ 146.3 m^2^ g^−1^	TiO_2_: 3.2; ZnIn_2_S_4_: 2.4	6030 μmol g^−1^ h^−1^ ^a^	300 W Xe lamp; 10 vol% TEOA; ZnIn_2_S_4_/TiO_2_ mass ratio 0.4; slurry-phase photocatalysis	[42]
1.5 wt.% CoTiO_3_/g-C_3_N_4_	Graphitic g-C_3_N_4_ and crystalline CoTiO_3_	S-scheme heterojunction interface	–	g-C_3_N_4_: 2.73; CoTiO_3_: 2.14	118 μmol g^−1^ h^−1^ ^b^	Visible-light irradiation; pure water; no sacrificial agent; optimized CoTiO_3_ content 1.5 wt.%	[43]
25 wt.% Co_3_Se_4_/TiO_2_	Anatase TiO_2_ and monoclinic Co_3_Se_4_	Co_3_Se_4_ microspheres decorating TiO_2_ nanosheets	–	–	6065 μmol g^−1^ h^−1^ ^a^	300 W Xe lamp; 20 vol% TEOA; optimized Co_3_Se_4_ loading 25 wt.%; slurry-phase photocatalysis	[44]
5 wt.% NiCoSe_2_/TiO_2_	Anatase TiO_2_ and hexagonal NiCoSe_2_	S-scheme heterojunction	–	–	4976 μmol g^−1^ h^−1^ ^a^	300 W Xe lamp; 20 vol% TEOA; optimized NiCoSe_2_ loading 5 wt.%; stability tested over five cycles	[45]
PCNNVs-L	Graphitic g-C_3_N_4_	3D porous hollow nanovesicles	Diameter ≈ 360 nm	2.78	10,300 μmol g^−1^ h^−1^ ^a^	Visible-light Xe lamp; aqueous TEOA; Pt cocatalyst; large-size PCNNVs-L configuration	[46]

^a^ Sacrificial-agent-assisted H_2_ evolution (not overall water splitting). ^b^ Pure water splitting without sacrificial agent.

#### 4.1.1. Synthesis Routes and Nanostructure Control

Different preparation routes provide distinct levels of control over the physicochemical characteristics of semiconductor photocatalysts. Sol–gel methods enable relatively homogeneous precursor mixing and compositional control, although hydrolysis, condensation, drying, and calcination conditions may considerably affect particle aggregation and crystallinity. Hydrothermal and solvothermal synthesis can promote the formation of well-defined nanoparticles, nanosheets, nanorods, or hierarchical structures by controlling solvent composition, temperature, pressure, reaction time, pH, and mineralizing agents. Precipitation and coprecipitation are widely used because of their operational simplicity and scalability, but precursor concentration, addition rate, aging, washing, and thermal treatment must be carefully controlled to avoid broad particle-size distributions or undesirable secondary phases [47,48,49].

Combustion synthesis can rapidly produce highly crystalline and porous materials, although the intense thermal conditions may lead to particle sintering or heterogeneous defect formation. Thermal polymerization is particularly relevant for carbon nitride-based photocatalysts, whose condensation degree, interlayer organization, surface functional groups, and defect concentration depend on the precursor and heating program. Photodeposition, impregnation, and deposition–precipitation are frequently employed to incorporate metallic or semiconductor cocatalysts [50,51]. Their effectiveness depends on achieving an appropriate loading, particle size, oxidation state, and surface dispersion without blocking light absorption or active sites. Likewise, the in situ growth of heterojunctions favors intimate contact between semiconductor components and may improve interfacial charge transfer. Template-assisted routes provide additional control over porosity and dimensionality, whereas immobilization and deposition onto glass, membranes, fibers, monoliths, or other supports facilitate catalyst recovery but may reduce the available surface area and alter photon and mass transport [50,52].

#### 4.1.2. Physicochemical Properties Relevant to Photocatalytic H_2_ Production

The properties produced during synthesis directly influence the sequential steps of photocatalytic hydrogen evolution. Crystalline phase and crystallinity affect carrier mobility, surface reactivity, and structural stability, while crystallite and particle size determine charge-diffusion distances, exposed surface area, aggregation behavior, and light scattering. Morphology and dimensionality—including zero-, one-, two-, and three-dimensional structures—modify the density of active sites, preferentially exposed facets, interfacial contact, and transport pathways. Surface area and porosity can increase reactant adsorption and cocatalyst dispersion, although a high surface area alone does not guarantee improved activity if the material contains excessive recombination centers [53,54,55].

The bandgap and optical absorption range determine the wavelengths that can generate electron–hole pairs, whereas conduction- and valence-band positions define the thermodynamic feasibility of the hydrogen and oxygen evolution reactions. Vacancies, dopants, and structural defects may extend optical absorption, introduce adsorption sites, and promote charge trapping; however, excessive or deep defects can act as non-radiative recombination centers [56]. In heterojunction systems, the quality of the interface, band alignment, internal electric field, and direction of charge transfer determine whether the coupled materials improve charge separation or introduce additional losses. Cocatalyst size, dispersion, loading, and oxidation state are also critical because they influence electron extraction, surface reaction kinetics, light shielding, cost, and long-term stability. Finally, resistance to photocorrosion and preservation of the crystalline and surface structure are required to ensure that high initial hydrogen production does not result from transient or unstable material transformations [56,57].

#### 4.1.3. Characterization Techniques and Structure–Performance Relationships

Complementary characterization is necessary to support the proposed relationship between nanostructure and photocatalytic performance. X-ray diffraction identifies crystalline phases and provides information on crystallinity and approximate crystallite size. SEM, TEM, and HRTEM reveal particle morphology, size, dimensionality, exposed structures, lattice fringes, and heterojunction interfaces, while EDS elemental mapping evaluates the spatial distribution of the constituent elements. Nitrogen adsorption–desorption analysis provides surface-area and porosity information, and UV–Vis diffuse-reflectance spectroscopy determines optical absorption behavior and supports bandgap estimation [58,59,60,61].

XPS is used to examine surface composition, oxidation states, chemical interactions, and defect-related species, whereas Raman and FTIR spectroscopy provide information on local bonding, lattice disorder, and surface functional groups. Photoluminescence and time-resolved photoluminescence can contribute to evaluating radiative recombination and carrier lifetimes, although their interpretation should be supported by additional evidence [25,62]. EPR is particularly useful for detecting paramagnetic defects and vacancy-related species. Mott–Schottky analysis provides information on semiconductor type and approximate flat-band potential, while electrochemical impedance spectroscopy and photocurrent measurements help evaluate interfacial resistance and charge-separation behavior. Finally, ICP-OES or ICP-MS can determine the actual amount of deposited dopant or cocatalyst. The combined use of these techniques enables a more reliable interpretation of hydrogen evolution by distinguishing material-related improvements from effects caused mainly by reactor geometry, irradiation, catalyst loading, reaction medium, or other operating conditions [63,64,65].

Table 14 provides a comparative overview of recently reported photocatalysts for hydrogen production, summarizing their synthesis routes, structural and morphological features, physicochemical properties, characterization evidence, photocatalytic performance, stability, and operating conditions. The selected systems include heterojunctions, metal-decorated semiconductors, doped photocatalysts, metal–organic frameworks, and metal-free materials designed to enhance visible-light absorption and charge-carrier separation. This comparison highlights the strong influence of catalyst composition, interfacial architecture, and reactor conditions on hydrogen evolution performance. However, direct comparison among the reported production rates should be made cautiously because catalyst loading, irradiation source, reaction medium, sacrificial agents, reactor configuration, and reporting units vary considerably among studies.

**Table 14:** Synthesis, physicochemical properties, photocatalytic performance, and operating conditions of selected photocatalysts.

Photocatalyst	Synthesis Route and Parameters	Structure and Morphology	Physicochemical Properties	Main Characterization Evidence	H_2_ Production and Stability	Reactor and Operating Conditions	Ref.
NiO/Ag/CdS 7% NiO + 0.5% Ag	CdS hydrothermal synthesis: 180 °C, 24 h. NiO precipitation at pH 8 and calcination at 300 °C for 2 h. Ag photodeposition using a 300 W Xe lamp.	CdS nanorods and NiO nanosheets. Ag nanoparticles (∼6 nm) at the CdS–NiO interface. Sandwich-type heterojunction.	$E_{g}\approx 2.4$ eV. BET: 24.7 m^2^ g^−1^. Ag surface plasmon resonance improves visible-light absorption and electron transfer.	XRD: hexagonal CdS and cubic NiO. ICP–OES: 0.47 wt.% Ag. SEM/TEM: intimate 1D/2D interface. XPS, PL, and EPR support a Z-scheme mechanism.	7.93 ± 0.13 mmol g^−1^ h^−1^. 25× CdS and 345× P25 TiO_2_. Activity retention: 88.1% after 9 h.	Quartz slurry reactor; 10 mg catalyst; 10 mL H_2_O + 2 mL TEOA; Ar purge for 30 min; 300 W Xe lamp; λ≥420 nm; 2 h cycles; GC analysis.	[66]
CdS–CoFe_2_O_4_/rGO 4 wt.% rGO	CdS and CoFe_2_O_4_ hydrothermal synthesis: 180 °C, 24 h. rGO obtained by chemical reduction of GO. Final impregnation: 80 °C, 6 h, pH 5.	Wurtzite CdS and spinel CoFe_2_O_4_. rGO sheets anchored onto the particles. Uniform nanocluster distribution.	$E_{g}$: CdS: 2.40 eV; CoFe_2_O_4_: 1.94 eV; composite: 2.05 eV. Improved charge separation.	XRD: CdS and CoFe_2_O_4_ phases. FTIR: Cd–S, Fe–O, Co–O, and rGO groups. DRS/PL: extended absorption and lower recombination. SEM/EDS: uniform distribution.	320,068 μmol g^−1^ h^−1^. Approximately 64× bare CdS. AQY: 20.5%. Improved cycling stability.	Double-walled Pyrex reactor; 150 mL; 50 ± 1 °C; N_2_ purge for 30 min; four 300 W LED lamps; λ≥420 nm; 0.5 M Na_2_S/Na_2_SO_3_; 3 h; GC-TCD.	[67]
2Cu–TiO_2_ SAS	Supercritical antisolvent micronization. TiO(acac)_2_ and CuAc in ethanol. 150 bar; 40 °C; 100 μm nozzle. Calcination: 450 °C, 2 h.	Uniform anatase nanoparticles (∼39 nm). Homogeneous Cu incorporation into the TiO_2_ lattice.	Reduced band gap relative to TiO_2_. Lattice expansion and oxygen vacancies. Enhanced visible-light absorption and charge separation.	XRD: anatase and Cu-induced peak shifts. FTIR/Raman: –OH groups and lattice distortion. FESEM/EDX: homogeneous Cu distribution. DRS/PL: lower recombination.	4040 μmol L^−1^ after 4 h. Normalized yield: 941 μmol H_2_ $g_{\mathrm{cat}}^{-1}$ ${\mathrm{mol}}_{\mathrm{LA}}^{-1}$ W^−1^.	Batch Pyrex reactor; 60 mL; 16 vol.% lactic acid; pH 2.1; 0.5–2 g L^−1^ catalyst; N_2_ purge; four 8 W lamps; 400–600 nm; TCD analysis.	[68]
0.13Ag/Au@CN	CN obtained by urea calcination: 550 °C, 3 h. Au and Ag sequential photodeposition. Optimal Ag loading: 0.13 wt.%.	CN nanosheets decorated with uniformly distributed spherical Au and Ag nanoparticles.	$E_{g}\approx 2.7$ eV. Metal co-decoration improves solar absorption, charge separation, and electron transport.	XRD: CN structure. XPS: metallic Au and Ag; Au-to-Ag electron transfer. PL: lower recombination. SEM/TEM: uniform nanoparticle distribution.	95.6× CN; 1.3× Au@CN; 9.8× Ag@CN. Activity decreased by a factor of 1.57 after four cycles.	Quartz reactor; 200 mL biodiesel wastewater; Ar purge; 500 W Xe lamp; 400 rpm; 40-fold dilution; 0.5 g L^−1^ catalyst; 10 vol.% TEOA; GC analysis.	[69]
NiS/borophene Ratio 2:1	NiS hydrothermal synthesis: 180 °C, 15 h. Borophene obtained by liquid exfoliation and calcination. Electrostatic self-assembly.	Borophene nanosheets (∼200 nm). Amorphous NiS spheres (∼1 μm). Intimate interfacial contact.	$E_{g}$: borophene: 1.52 eV; NiS: 1.67 eV; composite: 1.54 eV. Enhanced mesoporosity and charge separation.	XRD: crystalline borophene and amorphous NiS. XPS: Ni(II)/Ni(III), S^2−^, and B–B. SEM/TEM: intimate interface. DRS, PL, and DFT support a type-II mechanism.	51 μmol after 5 h. Approximately 174× bare borophene. Good structural stability.	Multichannel reactor; 25 °C; 10 mg catalyst; erythrosin B; 10 vol.% TEOA; 5 W LED; AM 1.5G; 100 mW cm^−2^; N_2_ purge; GC analysis.	[70]
Ce-BDC-DABCO N-doped Ce-MOF	Solvothermal synthesis. Ce(NO_3_)_2_·6H_2_O, H_2_BDC, and DABCO in water. 120 °C; 24 h.	Stable crystalline MOF. Well-defined nanosheets. DABCO improves structural stability and generates active defects.	Reduced band gap and enhanced visible-light absorption. Stable in saline water. H_2_ purity: ∼91%. Simultaneous LPG formation.	XRD: Ce-MOF structure. FTIR/Raman: Ce–BDC and Ce–DABCO coordination. BET/PL: surface area and charge separation. XPS: Ce^3+^ and C–N.	420.8 mmol h^−1^ g^−1^. Approximately 4× Ce-BDC. No sacrificial agent or noble metal. Stable in saline water.	Saline-water reactor; 3% salinity; 5 g L^−1^ catalyst; visible irradiation; 2.5 W cm^−2^; N_2_ purge; GC-TCD analysis.	[71]
O-4K-C_3_N_4_ O/K-codoped g-C_3_N_4_	Melamine calcination: 550 °C. K doping with KCl: 500 °C, 4 h. O codoping with ammonium oxalate and thermal treatment.	Exfoliated, porous, lamellar structure. K expands the interlayer spacing. O introduces defects and vacancies.	$E_{g}$: 2.75 to 2.39 eV. BET: 5.6 to 26.3 m^2^ g^−1^. Improved visible-light absorption and charge separation.	XPS: C, N, K, and O. XRD: interlayer expansion. SEM/TEM: porous morphology. FTIR/EPR: defects and –OH groups. DRS: absorption redshift.	682.5 μmol g^−1^ h^−1^. 7.7× g-C_3_N_4_. 1.6× K-doped material. No noble metals; good stability.	Aqueous suspension; white LED illumination; standard laboratory conditions; H_2_ quantified by gas chromatography.	[72]

### 4.2. Reactor Configuration

**Figure 4:**
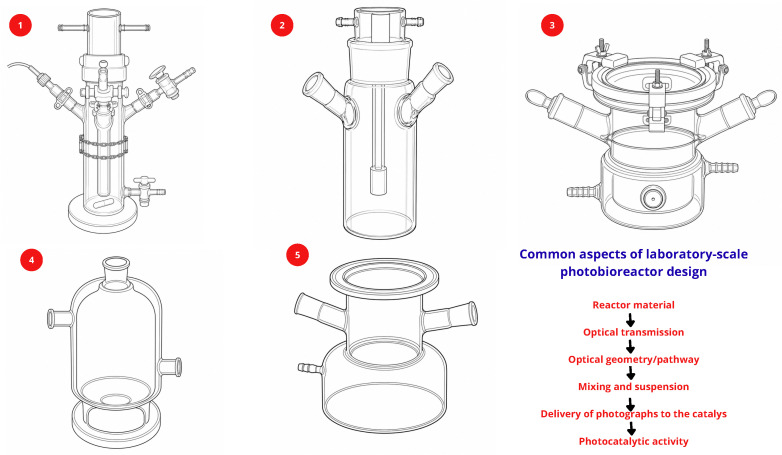
Typical configurations of laboratory reactors for photocatalytic water splitting, with emphasis on shape, optical geometry, and inlet and outlet arrangement. The reactor material, such as borosilicate glass or quartz, determines the optical transmittance and, therefore, the effective irradiation of the photocatalyst under visible or UV light.

The efficiency of photocatalytic water splitting processes depends not only on the electronic structure and surface properties of the semiconductor but also on reactor design and associated experimental conditions (Figure 4). Reactor engineering directly affects photon distribution, mass transfer, thermal management, and the quantification of gaseous products [73].

#### 4.2.1. Quartz and Three-Neck Glass Reactors with Cooling Jacket

At the laboratory scale, batch reactors remain the most widely used configuration for preliminary photocatalyst evaluation. These systems are typically constructed from borosilicate glass (Pyrex) or quartz, with quartz being preferred when high UV transmittance is required. The use of quartz enables direct irradiation of wide-bandgap materials without spectral attenuation caused by reactor wall absorption [73,74].

Laboratory reactors commonly incorporate multiple ports (functionally equivalent to three-neck configurations) dedicated to (i) inert gas purging, (ii) gas or liquid sampling, and (iii) instrumentation such as thermocouples or condensers. In advanced configurations, quartz tubes coated with TiO_2_ have been reported in fixed-bed arrangements, where catalyst immobilization mitigates sedimentation issues and simplifies material recovery [75,76].

Thermal control represents a critical parameter in batch systems. Continuous irradiation can induce temperature increases that alter surface kinetics and gas solubility, thereby affecting reproducibility. For this reason, jacketed reactors or immersed configurations with integrated thermal control have been developed to maintain quasi-isothermal operating conditions [77,78]. The literature consistently highlights that thermal stability is essential to distinguish purely photonic effects from unwanted thermal contributions.

#### 4.2.2. Closed Systems with Inert Gas Recirculation

For experiments requiring enhanced metrological reliability, particularly in hydrogen and oxygen quantification, closed-loop systems with recirculation are frequently employed. In these configurations, the reactor is connected to a circuit that includes a recirculation pump, an intermediate tank, and often a gas chromatography (GC) unit for continuous monitoring.

Paiu et al. (2025) [79] describe a continuous recirculation system in which the fluid flows from a feed reservoir through the photocatalytic reactor and returns to the storage tank, enabling stabilized operation and improved concentration homogeneity. This design enhances mass transfer and facilitates the removal of gaseous products.

In overall water splitting experiments, operation under an inert atmosphere (Ar or N_2_) is commonly adopted to remove dissolved oxygen and suppress reverse reactions that may consume the generated hydrogen. Gas recirculation maintains stable pressure conditions, improves product extraction, and minimizes measurement errors associated with leakage or uncontrolled gas accumulation. Gupta et al. (2022) [73] note that the configuration of gas extraction and analytical systems can significantly influence the reported productivity, underscoring the necessity of detailed experimental descriptions.

### 4.3. Typical Experimental Conditions

A recurrent trend in the literature is that the photocatalytic performance reported for hydrogen evolution is strongly conditioned by the experimental setup employed during evaluation, even when similar photocatalysts are used. Across recent studies, the most influential operational variables include the working volume of the reactor, catalyst loading, agitation regime, temperature control strategy, thermodynamic interpretation of hydrogen evolution, and the duration of inert gas purging before irradiation. These variables are often treated as secondary methodological details; however, the results surveyed indicate that they directly affect the reproducibility, apparent activity, gas quantification reliability, and comparability of reported hydrogen evolution rates [13,14,80].

**Figure 5:**
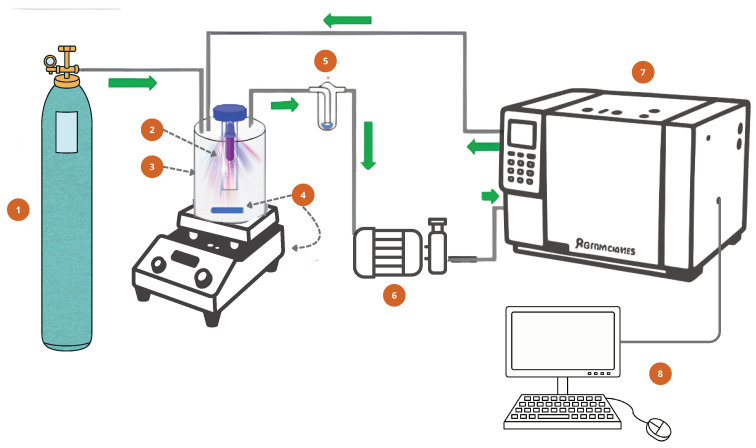
Experimental system for photocatalytic hydrogen production at laboratory scale. The setup consists of (1) carrier gas cylinder, generally N_2_, used to displace the O_2_ present in the system; (2) variable-volume cylindrical glass reactor, equipped with inlets and outlets for gas flow and recirculation; (3) UV or visible lamp placed in a central lamp holder or quartz tube, responsible for homogeneously irradiating the catalytic suspension; (4) hot plate and magnetic stirrer, used to keep the semiconductor suspended during the reaction; (5) moisture trap, containing an adsorbent material to remove moisture from the gas flow before analysis; (6) peristaltic pump, responsible for recirculating the gas throughout the system; (7) gas chromatograph, used to detect and quantify the produced H_2_; and (8) monitor or data acquisition system, where the experimental results are recorded, read, and processed [81].

In general, the reactor configuration can vary depending on the experimental design, reaction volume, type of irradiation, and how the photocatalyst is arranged, whether suspended or immobilized. However, regardless of the type of reactor used, some devices are essential for properly evaluating and quantifying hydrogen production, such as the carrier gas supply system, the irradiation source, the stirring or recirculation system, the moisture trap, the recirculation pump, the gas chromatograph, and the data acquisition system. Together, these components allow for the control of the reaction atmosphere, the transport of the generated gases, and the precise determination of the amount of H_2_ produced (Figure 5).

#### 4.3.1. Working Volume and Catalyst Concentration

The reviewed studies show that photocatalytic hydrogen evolution experiments are predominantly conducted at laboratory scale using small working volumes, generally between 50 and 500 mL, depending on reactor geometry and light source arrangement (Table 15). Smaller working volumes are frequently preferred because they improve light penetration, facilitate homogeneous suspension of the photocatalyst, and reduce the dead volume associated with gas sampling and purging. However, excessively low volumes may also increase the relative impact of evaporation, thermal drift, and headspace-related gas dilution effects [13,80].

**Table 15:** Influence of working volume and catalyst loading on photocatalytic H_2_ evolution experiments.

Parameter	Role in the Photocatalytic System	Experimental Implication	Recommended Reporting Information	Ref.
Working volume	Defines the amount of reaction medium contained in the reactor and interacts with reactor geometry, illuminated volume, optical path length, mixing, and gas accumulation.	Changes in working volume may modify light distribution, catalyst suspension, heat transfer, gas–liquid mass transfer, and the concentration of H_2_ measured in the headspace.	Report the total reactor volume, liquid working volume, illuminated volume, liquid depth, and reactor dimensions.	[13,80]
Catalyst concentration	Determines the amount of photocatalytic material available per unit volume and the balance between accessible active surface and light penetration.	Insufficient loading may limit the number of active sites, whereas excessive loading may increase turbidity, particle shielding, scattering, and photon attenuation. The optimum is system-dependent.	Report the concentration in g L^−1^, total catalyst mass, particle-deployment mode, working volume, and optical path length.	[14,80]
Total catalyst mass	Represents the absolute amount of photocatalyst used in the experiment and is frequently employed to normalize the H_2_ evolution rate.	Mass-normalized rates may lead to misleading comparisons when studies use different reaction volumes, illuminated areas, catalyst concentrations, or irradiation conditions.	Report total H_2_ production together with mass-normalized, volumetric, or area-based productivity whenever the available data permit it.	[13,80]
Headspace-to-liquid ratio	Defines the gas accumulation and sampling volume relative to the liquid reaction volume.	May influence gas dilution, pressure variation, gas–liquid equilibrium, sampling sensitivity, and the calculation of the total amount of evolved H_2_.	Report the headspace volume, liquid volume, operating pressure, sampling volume, sampling frequency, and gas-recirculation conditions.	[14]
Optical depth	Represents the effective distance traveled by radiation through the photocatalytic medium and depends on reactor geometry and suspension properties.	A large optical depth may increase absorption and scattering near the irradiated wall while producing weakly illuminated or dark regions within the reactor.	Report the optical path length, catalyst concentration, illuminated area, lamp position, reactor material, and irradiation geometry.	[13]

*Note:* Working volume and catalyst loading should not be interpreted as independent parameters. Their effects depend on reactor geometry, optical path length, irradiated area, particle properties, mixing conditions, irradiation intensity, and gas-sampling configuration. Consequently, no universal optimum volume or catalyst concentration can be established for all photocatalytic H_2_ evolution systems.

Catalyst loading is one of the most frequently varied parameters in photocatalytic hydrogen evolution experiments. In slurry systems, typical concentrations range from approximately 0.1 to 2.0 g L^−1^, although the exact optimum depends strongly on the optical properties of the catalyst, particle size, reactor depth, and irradiation intensity. The reviewed results consistently indicate the existence of an optimum loading range: below this threshold, the number of active sites is insufficient to maximize hydrogen production, whereas above it, excessive solid concentration leads to increased light scattering, particle shielding, and attenuation of photon penetration through the suspension [13,80].

This trend is particularly relevant because the hydrogen evolution rate is often normalized per gram of catalyst, which can create misleading interpretations if the total catalyst mass is varied without considering the corresponding optical limitations. In practical terms, a higher catalyst mass does not necessarily translate into a higher volumetric hydrogen productivity, and in many cases, excessive loading may reduce the apparent efficiency of photon utilization. Therefore, the literature suggests that catalyst concentration should be interpreted not as a purely compositional parameter but as an optical–reactive balance between available active surface and effective photon access [13,14].

Overall, the reviewed evidence indicates that the combination of working volume and catalyst loading should not be selected independently. Instead, these parameters must be co-optimized according to the irradiated area, optical path length, and sampling strategy in order to ensure that the measured hydrogen evolution reflects the real catalytic response rather than an artifact of the reactor configuration.

#### 4.3.2. Stirring and Thermal Control

Agitation is another operational variable that appears systematically across the reviewed studies, particularly in slurry-based photocatalytic systems (Table 16). In most experimental setups, magnetic stirring is employed to maintain catalyst suspension, minimize particle sedimentation, homogenize local irradiation exposure, and improve the transfer of dissolved species and evolved gas bubbles. Typical stirring rates reported in the literature range from approximately 300 to 1000 rpm, although in many studies this parameter is either vaguely described or omitted altogether [13,80].

The results reported in the literature suggest that insufficient agitation can lead to local concentration gradients, catalyst settling, and poor reproducibility in hydrogen evolution measurements. By contrast, adequate stirring improves phase contact and reduces the persistence of hydrogen bubbles adhered to catalyst particles or reactor walls, which may otherwise block active sites and locally alter light propagation. However, excessively vigorous stirring may also introduce secondary effects such as vortex formation, enhanced gas stripping, or perturbation of the optical field, especially in small-volume reactors [13,28].

Temperature control also emerges as a critical but often underestimated factor. Although photocatalytic hydrogen evolution is generally described as a light-driven process, many laboratory-scale systems experience non-negligible heating during irradiation, particularly when Xe or Hg lamps are used. As a result, jacketed reactors, external water circulation systems, or air-cooling arrangements are frequently employed to maintain the reaction medium close to room temperature, typically between 20 and 30 °C [19,80].

This is particularly important because temperature fluctuations can influence gas solubility, reaction kinetics, catalyst surface chemistry, and even the apparent rate of hydrogen evolution. In some studies, a modest increase in temperature is associated with improved kinetics and faster interfacial charge transfer; however, this does not necessarily imply improved photocatalytic efficiency, since part of the measured enhancement may originate from thermally assisted effects rather than purely photonic activation [13,14].

Taken together, the reviewed studies indicate that agitation and thermal management should be considered integral parts of the photocatalytic reaction environment rather than merely auxiliary control variables. Their influence becomes particularly relevant when comparing reported hydrogen production rates between different laboratories or reactor configurations.

**Table 16:** Key thermodynamic, energetic, hydrodynamic, and thermal aspects in photocatalytic H_2_ evolution.

Category	Parameter	Typical Range/Relevance	Common Practice/Issue	Implication/ Interpretation	Ref.
Thermo/energetic	Reaction medium	Defines redox env.; distinguishes overall splitting vs. assisted H_2_	Often simplified as “water splitting”	Separate stoichiometric splitting from sacrificial-agent-assisted	[21,80]
Thermo/energetic	Sacrificial agent	Facilitates hole consumption; alters oxidation half-reaction	Often underreported or treated as secondary	Report type/concentration; analyze separately	[13,80]
Thermo/energetic	Photon–H_2_ relation	Indicates photonic significance of H_2_ yield	Rates often given w/o photon normalization	Include AQE, AQY, EQE, or STH efficiency	[14,19]
Thermo/energetic	Temp/pH	Affect band alignment, kinetics, solubility, stability	Often not analyzed jointly with H_2_ data	Evaluate together with rate, stability, irradiation	[14,19]
Thermo/energetic	Gas composition	Impacts equilibrium, dissolved gases, back reactions	Purge gas/headspace often insufficiently reported	Specify purge type, time, headspace, atmosphere	[13]
Hydro/thermal	Stirring speed	Typically 300–1000 rpm in slurry reactors	Magnetic stirring commonly used	Ensures homogeneity and reproducible irradiation	[13,80]
Hydro/thermal	Mixing mode	Continuous mixing during irradiation	Mixing intensity/configuration often omitted	Improves mass transfer and catalyst exposure	[28]
Hydro/thermal	Thermal control	Reaction temp usually 20–30 °C	Water jackets or recirculation systems used	Minimizes lamp heating; separates thermal effects	[19,80]
Hydro/thermal	Cooling system	Water/air cooling applied externally	Config/variation not always reported	Improves stability, reproducibility, comparability	[13,14]
Hydro/thermal	Gas-bubble release	Depends on agitation, geometry, gas intensity	Bubble accumulation may block active sites	Mixing aids disengagement, reduces blocking	[28]

*Note:* AQE = apparent quantum efficiency; AQY = apparent quantum yield; EQE = external quantum efficiency; STH = solar-to-hydrogen efficiency. Ranges are representative of lab-scale photocatalytic H_2_ systems, not universally optimal.

#### 4.3.3. Thermodynamic Analysis of the Experimental System

In the reviewed literature (Table 16), thermodynamic analysis is not always reported explicitly; however, it remains essential for interpreting hydrogen evolution data correctly. In experimental photocatalytic systems, the thermodynamic relevance of the observed hydrogen production depends not only on the semiconductor band structure but also on the actual reaction medium, the presence or absence of sacrificial reagents, dissolved gases, operating temperature, and pH. As a result, the same hydrogen evolution rate may represent fundamentally different energetic scenarios depending on the reaction conditions employed [14,19].

A recurring issue in the literature is that many studies report hydrogen production rates without adequately distinguishing between true water splitting and hydrogen evolution assisted by sacrificial reagents. From a thermodynamic standpoint, these two situations are not equivalent. In the presence of alcohols, amines, or sulfide/sulfite systems, the oxidation half-reaction is substantially modified, and the energetic burden associated with water oxidation is reduced. Consequently, the hydrogen production measured under these conditions should be interpreted as *photocatalytic hydrogen evolution* rather than complete water splitting [21,80].

Another relevant thermodynamic consideration concerns the relationship between hydrogen evolution rate and energy input. Recent analyses emphasize that reporting only the amount of hydrogen evolved is insufficient for evaluating system relevance unless it is related to photon flux, irradiation spectrum, and energy consumption. Metrics such as apparent quantum efficiency (AQE), external quantum efficiency (EQE), and solar-to-hydrogen efficiency (STH) are therefore increasingly recognized as more informative descriptors than hydrogen production rate alone [14,19].

The reviewed evidence suggests that a thermodynamic interpretation of photocatalytic hydrogen evolution is essential not only for mechanistic understanding but also for ensuring that reported performance is discussed within a physically meaningful and comparable framework.

#### 4.3.4. Inert Gas Purging Time Before Irradiation

The purging step prior to irradiation is one of the most consistently reported pre-treatment procedures in photocatalytic hydrogen evolution experiments. Across the literature, inert gas purging is primarily used to remove dissolved oxygen, displace residual air from the reactor headspace, establish a controlled atmosphere, and prevent parasitic electron consumption by oxygen reduction. The gases most commonly employed are argon and nitrogen, with argon generally preferred in high-precision photocatalytic measurements due to its chemical inertness and lower risk of secondary interactions [13,80].

Typical purging times reported in the literature range from approximately 10 to 60 min, depending on reactor volume, gas flow rate, liquid volume, and headspace configuration. In small laboratory reactors, purge durations of 20–30 min are particularly common and are generally considered sufficient to establish oxygen-depleted conditions prior to illumination [13,80].

The results reviewed indicate that inadequate purging can substantially distort hydrogen evolution measurements. Residual dissolved oxygen may compete for photogenerated electrons, suppressing the observed hydrogen evolution rate and altering the apparent catalytic performance. Furthermore, incomplete removal of air from the headspace may compromise the accuracy of gas chromatographic quantification, particularly during the early stages of irradiation when hydrogen concentrations remain low [13,14].

Despite its importance, purging is often reported in a simplified manner without specifying gas flow rate, purge line configuration, or whether the liquid phase, headspace, or both were actively degassed (Table 17). This lack of detail limits reproducibility and makes it difficult to assess whether differences in reported hydrogen evolution rates are due to catalyst performance or to variations in pre-irradiation atmospheric conditioning [13,14].

**Table 17:** Typical inert gas purging conditions prior to photocatalytic H_2_ evolution tests.

Parameter	Typical Range/Practice	Main Function	Experimental Consequence	Ref.
Purge gas	Ar or N_2_	Displaces O_2_ from the reactor and headspace	Reduces parasitic electron consumption and side reactions	[13,80]
Purge time	10–60 min	Establishes an inert atmosphere before irradiation	Improves reproducibility of H_2_ evolution measurements	[13]
Common purge duration	20–30 min	Standard practice in bench-scale slurry systems	Provides sufficient degassing for routine photocatalytic tests	[14,80]
Headspace degassing	Frequently applied	Removes residual gases from the sampling volume	Improves reliability of gas analysis and H_2_ quantification	[14]
Dissolved O_2_ removal	Essential before irradiation	Prevents O_2_ from acting as an electron scavenger	Increases the measured H_2_ evolution rate	[13,80]

Overall, the literature indicates that the inert gas purging step should be regarded as a critical experimental condition rather than a routine preparatory detail. In photocatalytic hydrogen evolution studies, the reproducibility and reliability of the reported results depend substantially on the extent to which the initial gaseous environment is controlled before irradiation begins.

#### 4.3.5. General Discussion of Experimental Trends

Taken together, the reviewed results show that so-called “typical experimental conditions” are far from trivial methodological details. Instead, they exert a direct influence on hydrogen evolution rate, gas quantification reliability, optical efficiency, and inter-study reproducibility. Across the surveyed literature, the most consistent trends suggest that photocatalytic hydrogen evolution is generally favored under moderate working volumes, catalyst loadings within an optimum optical window, continuous stirring, controlled near-ambient temperature, a clearly defined reaction atmosphere, and sufficiently long inert gas purging prior to irradiation [13,14,80].

Importantly, these variables are strongly interdependent. For example, the optimal catalyst loading depends on reactor depth and mixing conditions; purge time depends on working volume and headspace; and temperature stability depends on the irradiation source and cooling strategy. Consequently, the reviewed evidence supports the view that photocatalytic hydrogen evolution should not be assessed solely on the basis of catalyst composition but rather as the result of a coupled material–reactor–operation system.

### 4.4. Influence of the Sacrificial Agent

**Figure 6:**
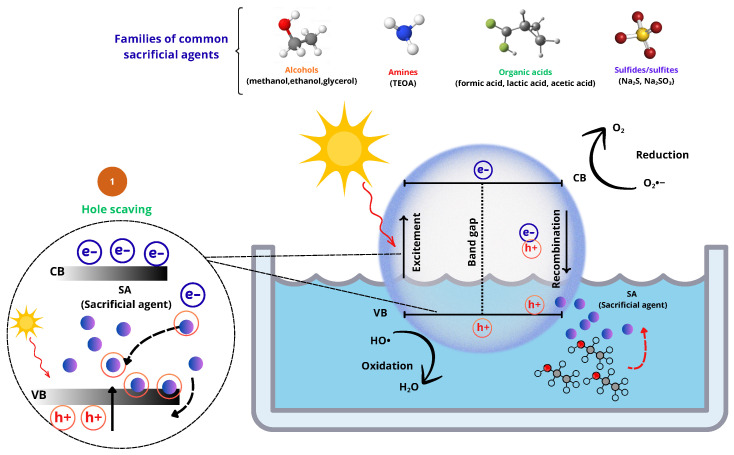
Schematic representation of the role of sacrificial agents in photocatalytic systems. Upon irradiation, the semiconductor generates electron–hole pairs; conduction-band electrons drive reduction reactions, while valence-band holes may either recombine or be scavenged by the sacrificial agent. This process modifies the oxidation half-reaction, suppresses charge recombination, and enhances photocatalytic efficiency. The response depends on the sacrificial agent properties, including donor ability, concentration, molecular structure, and interfacial adsorption. Therefore, H_2_ evolution in the presence of sacrificial agents represents an assisted reaction, not direct overall water splitting.

In photocatalytic $H_{2}$ production, the sacrificial agent should not be understood merely as an additive intended to consume holes; rather, it modifies the oxidation half-reaction and, in doing so, alters the overall kinetics, charge separation, and even the interpretation of photocatalytic performance itself. Therefore, $H_{2}$ evolution rates obtained in the presence of sacrificial agents should not be directly extrapolated as if they represented true overall water splitting (Figure 6) [82,83].

#### 4.4.1. Types of Sacrificial Agents

At present, studies show that the most common sacrificial agents can be grouped into the following categories: alcohols (e.g., methanol and ethanol), amines or alkanolamines such as triethanolamine (TEOA), organic acids such as lactic acid, and inorganic reducing systems such as sulfite/sulfide. This classification is relevant because each family exhibits a different oxidation propensity, a distinct surface interaction with the semiconductor, and a different influence on the dominant mechanistic pathway (Table 18) [84,85].

**Table 18:** Effect of sacrificial-agent type on photocatalytic H_2_ evolution under the reported experimental conditions.

Type	Sacrificial Agent	Concentration	Photocatalyst	H_2_ Production	Key Observation	Ref.
Alcohol	Methanol	20 vol.%	TiO_2_/Pt	High (relative)	Benchmark system	[86]
Alcohol	Ethanol	10 vol.%	g-C_3_N_4_/Pt	114 μmol g^−1^ h^−1^	Lower efficiency vs. TEOA	[87]
Amine	TEOA	10 vol.%	g-C_3_N_4_/Pt	810 μmol g^−1^ h^−1^	Strong hole scavenging	[87]
Organic acid	Lactic acid	10 vol.%	g-C_3_N_4_/Pt	427 μmol g^−1^ h^−1^	Favorable surface interaction	[87]
Alcohol	Methanol	15 vol.%	CdS/Pt	>1000 μmol g^−1^ h^−1^	High visible-light activity	[88]
Inorganic	Na_2_S/Na_2_SO_3_	0.25 M	CdS	>1500 μmol g^−1^ h^−1^	Highest efficiency reported	[89]
Biomass alcohol	Glycerol	15 vol.%	Au/TiO_2_	216 μmol g^−1^ h^−1^	Valorization potential	[90]
Biomass	Kelp (mannitol-rich)	1.5 g L^−1^	TiO_2_	Stable production	Sustainable feedstock	[91]
Alcohol	Ethylene glycol	10 vol.%	TiO_2_	200–400 μmol g^−1^ h^−1^	Improved stability	[92]
Amine	TEOA	10 vol.%	CdS	∼900 μmol g^−1^ h^−1^	Reduced recombination	[93]
Alcohol	Isopropanol	10 vol.%	ZnO	150–300 μmol g^−1^ h^−1^	Efficient oxidation	[94]
Organic acid	Formic acid	5–10%	TiO_2_	300–500 μmol g^−1^ h^−1^	Dual H_2_ source	[95]
Biomass	Glucose	10 g L^−1^	TiO_2_	100–200 μmol g^−1^ h^−1^	Kinetic limitations	[96]
Aromatic alcohol	Furfuryl alcohol	10 vol.%	ZnIn_2_S_4_/Pt	–	H_2_ + value-added product	[97]
Mixed system	MeOH/H_2_O	Variable	TiO_2_	Variable	Adsorption-dependent behavior	[86]

#### 4.4.2. Concentration *v*/*v*

The concentration of the sacrificial agent is a critically important variable because it determines the availability of electron donors, the efficiency of hole scavenging, and the competition for the active sites of the photocatalyst. Recent literature emphasizes that the selection of the sacrificial agent cannot be considered independently of its concentration, since both factors influence the overall efficiency and, in some cases, even the nature of the reported reaction. Therefore, the concentration should not be fixed by experimental convention but rather optimized for each catalytic system (Table 19) [84]. Moreover, not all recent systems report concentration on the same basis. In the case of miscible liquid donors, concentration is usually expressed in vol.%, whereas in biomass-derived systems it may be reported in g/L. This highlights that, in a review, it is essential to clearly distinguish among *v*/*v* concentration, molarity, and g/L, since direct comparison among them may lead to methodologically misleading interpretations [98].

**Table 19:** Effect of sacrificial-agent concentration on photocatalytic H_2_ evolution under the reported experimental conditions.

System	Sacrificial Agent	Concentration Range	Optimal Concentration	H_2_ Production	Trend	Ref.
g-C_3_N_4_/Pt	TEOA	5–15 vol.%	10 vol.%	810 μmol g^−1^ h^−1^	Maximum at intermediate concentration	[87]
TiO_2_/Pt	Methanol	5–25 vol.%	∼20 vol.%	High (relative)	Saturation at high concentration	[86]
CdS/Pt	Methanol	10–30 vol.%	15–20 vol.%	>1000 μmol g^−1^ h^−1^	Plateau beyond optimum	[88]
Au/TiO_2_	Glycerol	5–20 vol.%	15 vol.%	216 μmol g^−1^ h^−1^	Bell-shaped dependence	[90]
ZnO	Isopropanol	5–15 vol.%	10 vol.%	150–300 μmol g^−1^ h^−1^	Decline at higher concentration	[94]
TiO_2_	Ethylene glycol	5–15 vol.%	∼10 vol.%	200–400 μmol g^−1^ h^−1^	Improved stability near optimum	[92]
CdS	Na_2_S/Na_2_SO_3_	0.1–0.5 M	0.25 M	>1500 μmol g^−1^ h^−1^	Strong dependence on ionic strength	[89]
TiO_2_	Formic acid	2–10%	∼5–8%	300–500 μmol g^−1^ h^−1^	Optimum at moderate concentration	[95]
TiO_2_	Glucose	5–20 g L^−1^	∼10 g L^−1^	100–200 μmol g^−1^ h^−1^	Limited by mass transfer	[96]
TiO_2_	Kelp extract	0.5–2 g L^−1^	1.5 g L^−1^	Stable production	Optimum defined by organic composition	[91]
ZnIn_2_S_4_/Pt	Furfuryl alcohol	5–15 vol.%	∼10 vol.%	Dual production	Trade-off between H_2_ and oxidation products	[97]
CdS	TEOA	5–20 vol.%	10 vol.%	∼900 μmol g^−1^ h^−1^	Reduced recombination at optimum	[93]
MoS_2_/α-Fe_2_O_3_	TEOA	5–15 vol.%	∼10 vol.%	600–900 μmol g^−1^ h^−1^	Strong dependence on interfacial kinetics	[99]
TiO_2_	MeOH/H_2_O mixture	variable	system-dependent	variable	Controlled by adsorption equilibrium	[86]

As a concluding remark, it can be stated that there is no universally optimal *v*/*v* concentration of sacrificial agent for photocatalytic H_2_ production. Rather, the maximum activity is system-specific and arises from the interplay among the chemical nature of the sacrificial agent, the intrinsic properties of the photocatalyst, the characteristics and loading of the cocatalyst, the irradiation conditions, and the adsorption and charge-transfer processes taking place at the catalyst–solution interface. Under such conditions, apparent increases in activity may not reflect an intrinsic catalytic superiority but rather changes in surface adsorption, interfacial reaction pathways, cocatalyst-mediated charge separation, or photon utilization under different illumination regimes [84,98,100].

### 4.5. Influence of pH

**Figure 7:**
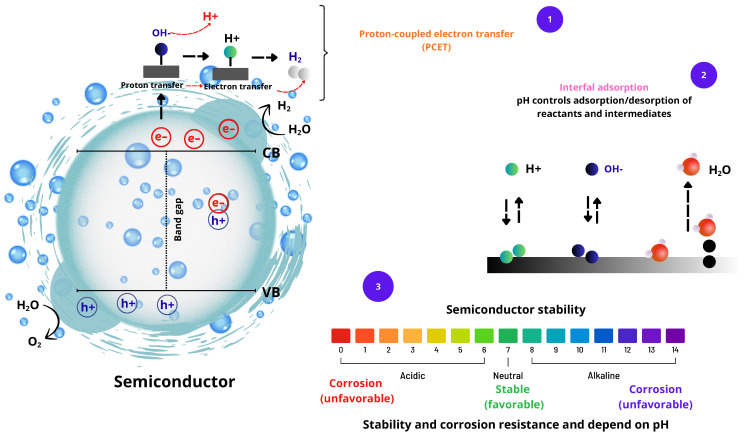
Influence of pH on the photocatalytic water splitting process over semiconductor materials. The pH modifies the availability of H^+^ and OH^−^ species, the adsorption of reactants and intermediates, proton–electron transfer, and the surface stability of the photocatalyst, directly affecting H_2_/O_2_ evolution and the possible corrosion of the material.

pH should be regarded as a first-order operational variable in photocatalysis, rather than merely as an auxiliary parameter of the reaction medium (Figure 7). Its influence arises from the availability of protonic and hydroxyl species, the surface charge state of the semiconductor, the acid–base equilibrium of surface hydroxyl groups, and the energetic alignment between the solid and the redox potentials of the solution. Therefore, the photocatalytic response to pH cannot be interpreted solely in terms of a higher proton concentration or a higher OH^−^ concentration but rather as the result of a combined modification of the interfacial kinetics and the effective energetic structure of the semiconductor/electrolyte system (Table 20) [101,102].

**Table 20:** Representative observations of pH-dependent H_2_ production in photocatalytic water-splitting systems.

Photocatalyst	pH Range	Optimal pH	Medium	Buffered	Reported H_2_ Production	Key Observation	Ref.
Ag–La–CaTiO_3_	2–12	4 and 10	Acidic/alkaline	No	High (dual maxima)	Activity peaks in both acidic and basic regimes	[103]
g-C_3_N_4_/Pt	3–11	∼7	Neutral	Yes/No	Moderate–high	Strong dependence on surface protonation	[87]
TiO_2_/Pt	2–10	6–7	Near-neutral	Yes	Stable	Band-edge alignment sensitive to pH	[104]
CdS	3–12	∼9	Alkaline	No	High	Enhanced hole scavenging in alkaline media	[93]
ZnO	4–11	∼8	Slightly alkaline	No	Moderate	Stability improves in alkaline conditions	[94]
MoS_2_/α-Fe_2_O_3_	4–10	∼7–8	Neutral–alkaline	No	High	Interfacial charge transfer influenced by pH	[99]
BiVO_4_	3–9	∼7	Neutral	Yes	Moderate	Surface states affected by pH	[105]
TiO_2_ (formic acid system)	2–8	∼4–5	Acidic	No	300–500 μmol g^−1^ h^−1^	Proton availability enhances H_2_ evolution	[95]
TiO_2_ (biomass system)	5–9	∼7	Neutral	No	Low–moderate	Local pH gradients affect activity	[91]
CdS/Pt	5–11	∼8–9	Alkaline	No	>1000 μmol g^−1^ h^−1^	Reduced recombination at higher pH	[88]
ZnIn_2_S_4_/Pt	4–10	∼7	Neutral	No	Moderate–high	Trade-off between HER and oxidation reactions	[97]
TiO_2_ (methanol system)	3–10	variable	Acidic–neutral	No	Variable	Strong dependence on adsorption equilibrium	[86]

*Note:* Reported H_2_ production values are not directly comparable because the studies employed different catalyst loadings, irradiation conditions, sacrificial agents, reactor configurations, reaction times, and normalization criteria. NR: not reported.

#### 4.5.1. Acidic, Neutral, and Alkaline Conditions

It is important to emphasize that experiments conducted under acidic, neutral, and alkaline conditions remain necessary, as there is no universal pH behavior applicable to all photocatalysts. For instance, in an Ag–La–CaTiO_3_ system operated under visible light, maximum $H_{2}$ production was reported at both pH 4 and pH 10, demonstrating that the same material can exhibit high performance in both acidic and basic regimes depending on changes in surface adsorption, charge transfer, and the involved redox pathways [106]. On the other hand, neutral conditions should not be regarded as a trivial case. Recent studies have shown that, even at pH values close to 7, the interfacial behavior can vary significantly depending on whether the medium is buffered or not. In a non-buffered neutral electrolyte, local pH gradients may develop during the reaction, whereas a buffered phosphate medium can suppress these gradients and better stabilize the semiconductor/electrolyte interface (Table 21). Therefore, when experiments are reported under “neutral pH” conditions, it is methodologically important to specify whether the system was buffered [107].

**Table 21:** Influence of acidic, neutral, and alkaline conditions on photocatalytic H_2_ production.

Photocatalyst	pH Range	Optimal Condition	Medium	Reported H_2_ Production	Key Observation	Buffered	Ref.
Ag–La–CaTiO_3_	2–12	Acidic/alkaline	Dual	High	Dual maxima at pH 4 and 10	No	[103]
g-C_3_N_4_/Pt	3–11	Neutral	Neutral	Moderate–high	Surface protonation dependent	Yes/No	[87]
TiO_2_/Pt	2–10	Near-neutral	Neutral	Stable	Optimal band alignment	Yes	[104]
CdS	3–12	Alkaline	Basic	High	Improved hole scavenging	No	[93]
ZnO	4–11	Alkaline	Basic	Moderate	Stability enhanced	No	[94]
BiVO_4_	3–9	Neutral	Neutral	Moderate	Surface states stabilized	Yes	[105]
MoS_2_/α-Fe_2_O_3_	4–10	Neutral–alkaline	Mixed	High	Charge transfer optimized	No	[99]
TiO_2_ (formic acid)	2–8	Acidic	Acidic	300–500	Proton-assisted HER	No	[95]
CdS/Pt	5–11	Alkaline	Basic	>1000	Reduced recombination	No	[88]
ZnIn_2_S_4_/Pt	4–10	Neutral	Neutral	Moderate–high	Balance HER/oxidation	No	[97]
TiO_2_ (methanol)	3–10	Variable	Acidic–neutral	Variable	Adsorption-controlled	No	[86]
Fe_2_O_3_	6–13	Alkaline	Basic	Moderate	CB shift improves reduction	No	[108]
WO_3_	1–7	Acidic	Acidic	Moderate	VB-driven oxidation favored	No	[109]
NiO	8–14	Alkaline	Basic	Moderate	p-type behavior enhanced	No	[110]
Cu_2_O	5–10	Neutral–alkaline	Mixed	Moderate	Surface stability limits range	No	[111]
SrTiO_3_	4–12	Neutral	Neutral	Moderate	Classical oxide behavior	No	[112]
BiOI	4–9	Neutral	Neutral	Moderate	Carrier separation affected	No	[113]

*Note:* Reported H_2_ production values are not directly comparable because the studies employed different catalyst loadings, irradiation conditions, sacrificial agents, reactor configurations, reaction times, and normalization criteria. NR: not reported.

#### 4.5.2. Influence of Synthesis pH on Nanostructure, Bandgap, and Photocatalytic Behavior of Semiconductor Photocatalysts

In physicochemical terms, pH is a key variable in semiconductor photocatalysis because it can influence both the interfacial energetics during the reaction and the structural properties established during material synthesis. In oxide semiconductors, recent studies indicate that band energies should not be treated as fixed intrinsic properties of the material, since they can shift with pH, electrolyte composition, and surface chemistry. Therefore, the effective positions of the conduction band and valence band depend on the actual chemical environment in which the semiconductor operates, rather than solely on its idealized bulk properties [102].

For operating photocatalytic or photoelectrochemical systems, pH modifies the protonation and deprotonation equilibria of surface hydroxyl groups and can induce shifts in the flat-band potential. Under near-ideal conditions, this displacement may approach Nernstian behavior, commonly approximated as ∼59 mV per pH unit. This dependence is important because it alters the redox driving force available for reactions such as H_2_ and O_2_ evolution and affects the interpretation of energetic alignment between the semiconductor and the electrolyte [107]. Moreover, the relevant pH is not always only the bulk value measured in solution. Local pH gradients may develop near the semiconductor/electrolyte interface during reactions, especially in non-buffered media, shifting the flat-band potential and disrupting ideal band-edge pinning. Buffered neutral electrolytes can suppress these gradients and preserve a more ideal interfacial behavior [107].

However, pH also plays a second and distinct role when it is used as a synthesis parameter. In this case, pH does not merely alter the energetic alignment during operation; it determines how semiconductor nanostructures are formed. The synthesis pH can regulate precursor hydrolysis, condensation, precipitation, nucleation, crystal growth, phase formation, exposed facets, aggregation, defect density, surface area, and optical bandgap. As a consequence, the photocatalytic response of a semiconductor may arise not only from pH-dependent band-edge shifts in the reaction medium but also from pH-induced changes in the nanostructure produced during synthesis.

This distinction is relevant for nanostructured photocatalysts because size, morphology, surface area, and defect chemistry strongly affect light absorption, charge separation, surface adsorption, and recombination pathways. For example, TiO_2_ nanoparticles prepared under different pH conditions show pH-dependent particle size, surface area, bandgap, surface hydroxylation, pH_PZC_, and methylene blue degradation efficiency [114]. Similarly, acidic sol–gel synthesis of TiO_2_ can modify anatase/rutile phase composition, crystallite size, morphology, and BET surface area [115]. In ZnO nanoparticles obtained by green synthesis, the reaction pH affects nanoparticle size, crystallinity, bandgap, and methyl orange degradation performance [116,117]. In mixed oxide systems such as ZnO/CuO, pH-dependent precipitation controls the formation of nanorods, nanosheets, urchin-like structures, or flower-like architectures, which directly modifies visible-light photocatalytic activity [118].

A similar pH-directed nanostructuring effect has been reported for bismuth-based photocatalysts. In BiOI, increasing the synthesis pH reduces crystallite size, changes the dominant exposed facets from {001} to {110}, increases the surface area, and slightly modifies the optical bandgap, producing different trends in weight-normalized and surface-area-normalized photocatalytic activity [119]. In γ-Bi_2_MoO_6_, an optimum synthesis pH favors pure-phase formation, visible-light absorption, and enhanced methylene blue degradation [120]. Likewise, pH-regulated assembly of Sb_4_O_5_Cl_2_ controls the transition from particle assemblies and hollow microspheres to individual microbelts, producing changes in surface area, bandgap narrowing, charge-transfer resistance, and photocatalytic performance [121]. These examples show that synthesis pH is a practical tool for engineering nanoscale semiconductor properties.

Therefore, pH should be considered at two complementary levels in photocatalytic systems. First, the reaction pH affects redox potentials, surface charge, adsorption, semiconductor/electrolyte interfacial energetics, and band-edge alignment. Second, the synthesis pH determines the nanostructural and optical properties of the photocatalyst before it is introduced into the reactor. Neglecting this distinction may lead to misleading interpretations, since changes in photocatalytic behavior could be incorrectly attributed only to band-edge shifts, even when they originate from differences in particle size, morphology, surface area, defect concentration, or bandgap generated during synthesis [101,102]. Representative examples of pH-controlled nanostructuring in semiconductor photocatalysts are summarized in Table 22.

**Table 22:** Influence of synthesis pH on particle size, morphology, bandgap, and photocatalytic behavior of semiconductor photocatalysts.

Photocatalyst	Synthesis Route and pH Range	Particle Size and Morphology	Physicochemical Properties	Main Characterization Evidence	Photocatalytic Behavior	Main pH-Related Interpretation	Ref.
TiO_2_ nanoparticles	Hydrothermal/sol–gel preparation under adjusted pH values: 1.6, 7.0, and 10.	Semispherical TiO_2_ nanoparticles. Particle size decreased with increasing pH: 12.4 nm at pH 1.6, 10.5 nm at pH 7.0, and 8.7 nm at pH 10. Agglomeration decreased at higher pH.	BET surface area increased with pH: 12.4, 119.4, and 183.6 m^2^ g^−1^ for pH 1.6, 7.0, and 10, respectively. $E_{g}$ decreased from 3.54 to 3.47 and 3.40 eV.	TEM: particle size and morphology. N_2_ adsorption: surface area and pore structure. UV–vis/Tauc: bandgap. XPS: surface hydroxyl groups. Zeta potential: pH_PZC_.	MB degradation increased from 73% to 93% and 97%. The highest degradation rate was obtained for TiO_2_ prepared at pH 10.	Increasing synthesis pH reduced particle size and agglomeration, increased surface area and surface hydroxylation, and improved electrostatic interaction with the cationic dye. This case shows that pH can tune the photocatalyst through morphology and surface charge, not only through band-edge effects.	[114]
TiO_2_ nanoparticles	Sol–gel synthesis from titanium(IV) isopropoxide. Acidic precursor solutions adjusted to pH 2, 4, and 6. Calcination: 500 °C for 5 h.	Crystallite size decreased as pH increased: 33 nm for TiO_2_-2, 16 nm for TiO_2_-4, and 14 nm for TiO_2_-6. TiO_2_-4 and TiO_2_-6 showed smoother and more agglomerated surfaces.	TiO_2_-2 contained mainly anatase with minor rutile. TiO_2_-4 and TiO_2_-6 showed single anatase phase. BET surface areas increased slightly: 50, 54, and 59 m^2^ g^−1^.	XRD: anatase/rutile phase composition and crystallite size. Raman: phase confirmation. SEM: surface morphology. BET: surface area. XPS and FTIR: surface composition.	Photocatalytic activity was not directly evaluated in this study. The work supports the structural effect of synthesis pH on TiO_2_ formation.	Acidic synthesis pH controls hydrolysis, condensation, crystallite growth, and phase formation in TiO_2_ sol–gel synthesis. Higher pH within the acidic range favored smaller crystallites and slightly higher surface area.	[115]
TiO_2_ with Ti^3+^ and oxygen vacancies	Low-temperature sol–gel synthesis. pH values: 0.6, 1.0, 1.9, and 4.7. Calcination temperatures from 300 to 700 °C. Representative optimum: TN-1.0-300.	Anatase TiO_2_ nanoparticles. pH and calcination temperature controlled grain size, crystallinity, surface hydroxylation, and hydrophilicity.	Ti^3+^ and oxygen vacancies narrowed the bandgap and enhanced visible-light absorption. TN-1.0-300 showed favorable surface area, defect structure, and exposed crystal planes.	XRD: anatase structure and grain size. XPS/EPR: Ti^3+^ and oxygen vacancies. BET: surface area and pore structure. UV–vis DRS: visible absorption and bandgap. Contact angle: hydrophilicity.	TN-1.0-300 showed the best MO degradation under simulated sunlight and visible light. Reported first-order rate constants: 0.182 min^−1^ under simulated sunlight and 0.081 min^−1^ under visible light.	Optimum acidic pH partially controlled TBOT hydrolysis and condensation, generating Ti^3+^ and oxygen-vacancy defects. These defects improved visible-light response, ROS generation, and electron–hole separation.	[122]
ZnO nanoparticles	Green synthesis using *Portulaca oleracea* leaf extract. pH values: 4, 6, 9.5, and 11. Calcination: 500 °C for 2 h.	Crystallite sizes: 27.38 nm at pH 4, 26.04 nm at pH 6, 25.39 nm at pH 9.5, and 22.17 nm at pH 11. Mostly spherical or oval ZnO nanoparticles with some agglomeration.	Direct optical bandgap: 2.97 eV at pH 4, 3.69 eV at pH 6, 3.90 eV at pH 9.5, and 3.97 eV at pH 11. Urbach energy decreased from 0.395 to 0.137 eV.	XRD: hexagonal ZnO phase and crystallite size. SEM: particle morphology. FTIR: plant-extract functional groups and Zn–O vibration. UV–vis/Tauc: direct and indirect bandgaps. Urbach analysis: structural disorder.	MO degradation was evaluated under solar irradiation. Most of the MO dye was decomposed after 120 min, with behavior dependent on synthesis pH.	H^+^/OH^−^ concentration influenced ZnO nucleation, growth, crystallinity, particle size, and bandgap. Higher pH reduced crystallite size and widened the optical bandgap, although excessive pH may favor aggregation or impurities.	[116]
ZnO nanoparticles	Green synthesis using *Allium caliphalum* Wendelbow leaf extract. Growth solution pH: 7–12.	SEM particle size ranged from approximately 30 to 72 nm. XRD crystallite size ranged from 15 to 24 nm. pH 8 yielded smaller and more uniform nanoparticles.	Bandgap energy varied from approximately 3.25 to 3.14 eV. pH 8 showed the highest bandgap (∼3.25 eV), while pH 12 showed a lower bandgap (∼3.14 eV).	SEM: particle size and morphology. XRD: crystallite size and crystallinity. UV–vis: bandgap. FTIR: plant-derived stabilizing groups. EDX: elemental composition.	The ZnO sample prepared at pH 8 showed the highest MO degradation efficiency: 74% in 140 min under UV irradiation.	Moderately alkaline pH promoted balanced nucleation and growth, producing uniform particles with favorable crystallinity. Very high pH promoted fast precipitation, aggregation, larger particles, and lattice defects.	[117]
ZnO/CuO nanocomposites	Chemical bath precipitation. pH values: 1.5, 3.5, 6.8, 10, and 11. pH adjusted with HNO_3_ or NH_4_OH.	pH 1.5: no distinct ZnO/CuO nanostructures. pH 3.5: ZnO nanorods and CuO nanosheets. pH 6.8: ZnO nanorods and CuO urchin-like morphology. pH 10: flower-like structures.	Bandgaps: 3.2 eV for ZC3.5, 2.9 eV for ZC6.8, and 3.0 eV for ZC10. pH also affected pore volume, oxygen vacancies, and charge recombination.	XRD: ZnO and CuO phase formation. FESEM/HRTEM: pH-induced morphology. HRXPS: surface chemical states and oxygen vacancies. BET: porosity. DRS/PL: bandgap and recombination.	After 150 min of visible-light irradiation, MB degradation was 71% for ZC3.5, 85.8% for ZC6.8, and 100% for ZC10.	Synthesis pH-controlled Zn(II)/Cu(II) speciation, hydroxide formation, and oxide growth. The resulting morphology modified exposed active facets, oxygen vacancies, recombination behavior, and visible-light photocatalytic response.	[118]
BiOI nanosheets	Hydrothermal synthesis. pH adjusted from 2.3 to 8 using NaOH.	Crystallite size decreased from 126 nm to 16 nm as pH increased from 2.3 to 7. Low-pH samples formed large {001}-facetted crystallites. Higher-pH samples formed thinner and smaller {110}-facetted platelets.	Surface area increased from 0.65–3.98 m^2^ g^−1^ at pH 2.3–4 to 9.8–23.9 m^2^ g^−1^ at pH 5–8. Bandgap increased slightly from 1.72 to 1.94 eV.	XRD: tetragonal BiOI and facet orientation. SEM/TEM: nanosheet morphology and exposed facets. BET: surface area. DRS: bandgap. Photodegradation tests: facet-dependent activity.	Weight-normalized *p*-cresol degradation increased from 0.28 h^−1^ g^−1^ at pH 2.3 to 3.65 h^−1^ g^−1^ at pH 6, then decreased at higher pH.	Synthesis pH-controlled BiOI precipitation, crystallite size, and exposed facets. The overall activity resulted from the balance between intrinsic facet reactivity and available surface area.	[119]
γ-Bi_2_MoO_6_ nanoparticles	Co-precipitation synthesis. pH values: 1.5, 3.0, and 5.0. Calcination: 475 °C for 5 h.	pH 3.0 produced pure γ-Bi_2_MoO_6_ nanoparticles with a single hexagonal morphology. pH 1.5 and 5.0 produced mixed phases and mixed morphologies.	Bandgaps: 2.59 eV for BM1, 2.48 eV for BM2, and 2.55 eV for BM3. All samples absorbed in the visible region.	XRD/Raman: phase purity and γ-Bi_2_MoO_6_ formation. FESEM/TEM: hexagonal and mixed morphologies. DRS: bandgap. PL: hydroxyl radical formation and photocatalytic mechanism.	MB degradation after 120 min: 87.87% for BM1, 95.44% for BM2, 91.28% for BM3. BM2 + H_2_O_2_ reached 98.89%.	An optimum synthesis pH of 3.0 favored pure γ-phase formation, suitable morphology, and visible-light absorption. Phase purity and morphology were more decisive than pH alone.	[120]
ZnCr_2_O_4_ nanoparticles	Co-precipitation method. pH values: 7, 11, and 14. Different alkaline agents and capping agents were compared.	pH, alkaline agent, and capping agent strongly affected purity, particle size, and morphology. NaOH at high pH promoted smaller particles compared with weaker alkaline agents. SDBS/NaOH favored fine ZnCr_2_O_4_ nanoparticles.	For the selected ZnCr_2_O_4_ sample: $E_{g}$ estimated by UV–vis: 3.35 eV. $E_{g}$ estimated by PL: 3.96 eV. UV–vis was considered the more accurate bandgap estimation.	FESEM: morphology and particle size. XRD: cubic spinel ZnCr_2_O_4_ and crystallite size. FTIR/EDS: bonding and composition. DRS/PL: bandgap. VSM: magnetic behavior.	Under UV irradiation for 30 min, degradation reached 95.26% for Eosin-Y and 13.42% for phenol red.	The pH and alkaline agent controlled nucleation and growth rates. Fast pH increase with NaOH favored nucleation over growth, producing smaller and purer ZnCr_2_O_4_ particles with photocatalytic activity.	[123]
Sb_4_O_5_Cl_2_ crystallites	Template-free hydrothermal synthesis. Precursor pH: 1, 2, 3, 4, and 5. Temperature: 160 °C for 12 h.	pH 1: particles assembled by irregular cuboids. pH 2: hollow microspheres assembled by irregular cuboids. pH 3–5: individual microbelts.	BET surface areas: 14.04, 18.62, 8.69, 8.63, and 8.45 m^2^ g^−1^ for pH 1–5. Bandgaps: 3.29, 3.28, 3.36, 3.37, and 3.38 eV.	XRD: monoclinic Sb_4_O_5_Cl_2_. SEM/TEM: pH-controlled assemblies and microbelts. XPS: electronic coupling. UV–vis DRS: bandgap and absorption edge. Mott–Schottky/EIS: carrier behavior and resistance.	pH 2 sample showed the best performance. RhB degradation rate constant: 0.08185. IPA oxidation also showed the highest acetone and CO_2_ evolution for pH 2.	At pH 2, particle assembly induced inter-particle electronic coupling, self-narrowed bandgap, higher surface area, enhanced charge separation, and lower electron-transfer resistance. This demonstrates that pH can tune photocatalysis by directing hierarchical assembly.	[121]

### 4.6. Irradiation Conditions

**Figure 8:**
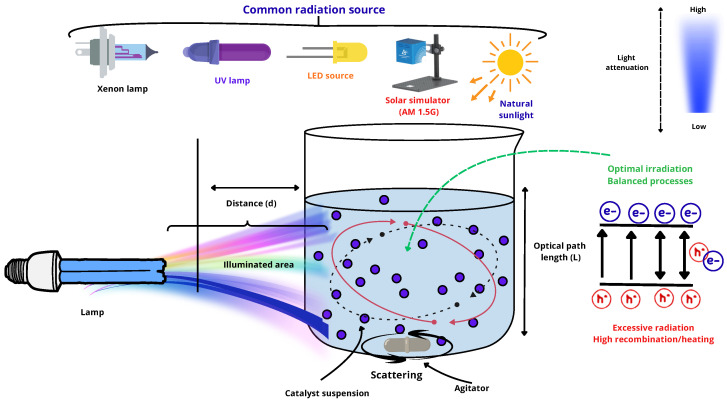
Effect of the irradiation source and optical path on photocatalytic efficiency. The lamp–reactor distance, incident intensity, scattering, and suspension turbidity determine the number of photons that reach the catalyst surface, influencing electron-hole pair generation, recombination, and photocatalytic activity.

Irradiation conditions are a critical factor in photocatalysis, as the observed activity depends not only on the material but also on the light source, its spectral distribution, the incident intensity, the reactor geometry, and the efficiency with which photons reach the catalyst’s active surface. For this reason, irradiation conditions are one of the most determining parameters in the evaluation of photocatalytic systems, since the observed activity depends not only on the intrinsic properties of the material but also on the type of light source. Table 23 describes the radiation sources used in different studies (Figure 8).

#### 4.6.1. Xe and Hg Lamps and AM 1.5G Solar Simulators

In laboratory-scale photocatalysis, xenon lamps, mercury lamps, UV/visible LEDs, and solar simulators are the most commonly used irradiation sources. Xenon lamps are widely employed because their continuous emission can approximate the solar spectrum when combined with AM 1.5G filters. Therefore, they are frequently used in studies involving hydrogen production, CO_2_ reduction, and pollutant degradation under simulated solar-light conditions. Typical configurations include Xe lamps of 250–300 W combined with visible–light cutoff filters, such as λ>420 nm or AM 1.5G filters to simulate global solar irradiation [132,133].

Mercury lamps, on the other hand, are mainly used when UV irradiation is required. Their main advantage is their strong emission in the UV region, particularly near 254, 313, 365, or 366 nm, depending on the type of lamp. However, they also present important drawbacks: they generate heat, may modify the temperature of the reaction medium, and often require cooling systems [129].

In contrast, UV-LEDs are increasingly being used because they allow the selection of specific wavelengths, intensity control through pulse-width modulation (PWM), and reduction of residual heat. A tubular LED reactor has been reported using 30 UV-LEDs at 367 nm, with a total radiant flux of 42 W and an electrical power consumption of 111 W. In addition, this system allows irradiation intensity adjustment and can accommodate tubular reactors with diameters up to 28 mm within an irradiated length of 300 mm. This type of design is relevant because it enables the study of light-distribution effects rather than considering only the nominal power of the lamp [129,134].

To simulate solar irradiation, the most widely used standard is AM 1.5G. The AM 1.5G spectrum represents a standard global solar irradiance of 1000 W m^−2^, equivalent to 100 mW cm^−2^, and is used as a reference for 1-sun conditions. In addition, the ASTM G173 spectrum is commonly used as a reference to represent terrestrial solar irradiance under defined atmospheric conditions. Experimentally, this means that a solar simulator must be calibrated with a radiometer or reference cell to ensure that the reaction area receives an irradiance close to 100 mW cm^−2^ when 1 sun is declared [135].

#### 4.6.2. Use of Optical Filters for UV, Visible, or Combined Radiation

The use of optical filters is essential in photocatalytic studies because it allows control over the spectral region incident on the material and makes it possible to distinguish the contribution of UV, visible, or simulated solar irradiation. This separation is particularly important for identifying whether the photocatalytic activity originates from direct semiconductor excitation, electronic defects, doping processes, plasmonic effects associated with metallic nanoparticles, or carbon-mediated mechanisms [136].

In visible-light experiments, cutoff filters with λ>420 nm are commonly used to remove the UV fraction of the light source and evaluate the response of the photocatalyst to lower-energy photons. This criterion is especially relevant when studying modified materials, heterostructures, or hybrid systems designed to extend light absorption into the visible region. For example, TiO_2_–HC systems have been reported for Cr(VI) reduction using a 250 W Xe lamp with a λ>420 nm filter, while CoFe_2_O_4_–C photocatalysts have also been evaluated under similar visible-light conditions. Likewise, some studies employ high-power mercury lamps combined with AM 1.5G filters to simulate solar irradiation or with 420 nm cutoff filters to restrict excitation to the visible region [136,137].

For UV irradiation, optical filters allow the selection of specific spectral regions, such as UVA, UVB, or UVC, depending on the objective of the experiment and the nature of the semiconductor. In wide-bandgap materials such as TiO_2_, ZnO, or SrTiO_3_, UV radiation is particularly relevant because their band gap values generally lie within the range of 3.0–3.4 eV. For this reason, light sources such as Hg lamps or UV-LEDs emitting near 365–367 nm can promote direct semiconductor excitation and generate electron–hole pairs responsible for photocatalytic redox reactions. In this context, UV-LED reactors operating at 367 nm are suitable configurations for evaluating UV-active semiconductors while also offering greater control over irradiation intensity and spatial light distribution [138,139].

On the other hand, when AM 1.5G filters are used, the purpose is not to isolate a specific spectral region but rather to reproduce, under controlled conditions, the global distribution of terrestrial solar radiation. This condition is particularly useful when discussing photocatalytic performance under scenarios closer to real solar applications. However, the term “simulated solar light” should be used with precision, since it is not sufficient to report only the type of lamp or filter employed. To ensure reproducibility and comparability among studies, it is necessary to report the incident irradiance, filter type, solar simulator model, illuminated area, distance between the light source and the reactor, and the calibration conditions of the irradiation system [140].

#### 4.6.3. Irradiation Intensity and Spectral Distribution

Irradiation intensity is a critical parameter in the evaluation of photocatalytic systems and should not be confused with the nominal power of the lamp. For example, a 300 W light source does not imply that this power directly reaches the catalyst surface. For comparison and reproducibility purposes, the relevant parameter is the incident irradiance on the active area of the reactor, which is commonly expressed in mW cm^−2^ or W m^−2^. It is also necessary to distinguish between the electrical power consumed by the source, the emitted radiant flux, the radiation incident on the reactor, and the fraction of light effectively absorbed by the photocatalyst [141].

A representative example of rigorous characterization is the tubular UV-LED reactor reported by Herrmann et al. Although the system has a maximum radiant flux of 42 W, the authors did not limit their analysis to this value; instead, they also determined the actual electrical power consumption and the irradiance inside the reactor. At a radiant flux of 12.6 W, the electrical consumption was 44.0 W, whereas at 42 W it reached 131.2 W, with wall-plug efficiencies of 29.5 and 32.0%, respectively. In addition, irradiance was measured at different positions inside the reactor, with values above 10 mW cm^−2^ observed across a significant portion of the irradiated zone. This type of characterization enables a more accurate relationship to be established among the supplied energy, the available light, and the photocatalytic response of the system [142].

The spatial distribution of light is as relevant as the average irradiance. In a photocatalytic reactor, the presence of high-irradiance zones near the light source and regions with lower exposure can generate significant differences in the apparent activity of the catalyst. This effect is particularly important in tubular, fixed-bed, thin-film, or slurry reactors, where shadowing, scattering, reflection, and partial absorption of radiation by the reaction medium may occur. Therefore, reactor geometry and light-source arrangement should be considered fundamental experimental variables, rather than merely auxiliary elements of the setup [143].

In this regard, Herrmann et al. point out that when mercury lamps are replaced by LED arrays, the geometric distribution of the emitters becomes decisive for achieving homogeneous irradiation. This observation is consistent with previous studies on LED photocatalytic reactors, where a more uniform light distribution has been shown to increase photonic efficiency and improve the amount of pollutant removed per unit of energy consumed. Consequently, light-source optimization should not focus solely on increasing power, but rather on maximizing the effective utilization of photons within the active volume or surface of the reactor [129].

In fluidized-bed systems, light distribution also plays a decisive role in the overall process efficiency. The study by Wu, Gouda, and Ozin demonstrates that photofluidized reactors can improve light penetration and particle–photon interaction compared with fixed beds. Through CFD–DEM simulations coupled with ray tracing, the authors showed that fluidization promotes greater light absorption, especially for particles with low intrinsic absorptivity. However, system performance strongly depends on hydrodynamic conditions, since excessive gas flow can displace particles outside the illuminated zone and reduce process efficiency. Therefore, in this type of reactor, optimization must simultaneously consider irradiance, spectral distribution, bed dynamics, and the overlap between the illuminated region and the catalytically active zone [130,130].

#### 4.6.4. Laboratory Level Consumption vs. Real (Solar) Consumption

A key difference between laboratory photocatalytic tests and real solar operation is the energy source used for irradiation. In laboratory studies, artificial light sources such as Xe, Hg, or LED lamps require electrical power; therefore, high degradation efficiencies or H_2_ production rates obtained under 300 W, 500 W, or 1 kW lamps do not necessarily indicate energetic feasibility unless the results are normalized by electrical consumption, incident photon flux, or absorbed radiant energy [144].

The total laboratory energy demand may include not only the lamp but also the power supply, cooling system, stirring, pumps, controllers, and ventilation. This is particularly relevant for Xe and Hg lamps, which generate considerable heat and may require cooling to avoid thermal effects on the reaction. For this reason, reporting electrical consumption, radiant flux, irradiance, and wall-plug efficiency is essential to relate photocatalytic performance to the real energy cost of the process [145,146].

In real solar operation, irradiation does not require direct electrical input, but the process is affected by solar variability, cloud cover, incidence angle, atmospheric conditions, and geographical location. Thus, AM 1.5G irradiation at 100 mW cm^−2^ is useful as a standardized laboratory reference, but it does not fully reproduce outdoor solar conditions or pilot-scale operation [132,133,147].

For scale-up, photocatalytic performance should be reported not only as degradation percentage or production rate but also through energetic and photonic indicators, such as apparent quantum efficiency, photonic efficiency, electrical energy per order of degradation, productivity per illuminated area, and H_2_ production normalized by catalyst mass, time, and incident power. These metrics allow more reliable comparisons between laboratory results and potential solar applications [148].

#### 4.6.5. Quantitative Comparison of Representative Photocatalytic Hydrogen Production Systems

To facilitate a more direct comparison among the reviewed studies, Table 24 summarizes representative photocatalytic H_2_ evolution systems using a common set of reactor, operational, and performance descriptors. The extracted parameters include reactor configuration, catalyst deployment mode, working volume, catalyst loading, sacrificial-agent composition, pH, irradiation source, optical filter, mass-normalized H_2_ evolution rate, volumetric or area-based productivity, and AQE/AQY when available. Catalyst loading was calculated from the reported catalyst mass and working volume only when both values were explicitly available. Parameters that could not be reliably recovered from the original reports are identified as NR rather than retrospectively estimated.

## 5. Discussion

Table 25 shows that photocatalytic H_2_ production is governed by the combined influence of semiconductor properties, reactor configuration, and operating conditions. Recent studies indicate a clear transition from conventional slurry-type batch reactors toward more advanced configurations designed to improve photon utilization, mass transfer, product separation, and process scalability [149,150,151]. This evolution reflects a broader recognition that the measured performance of a photocatalyst cannot be attributed exclusively to its intrinsic structural or electronic characteristics.

The quantitative evidence compiled in Table 13 and Table 18, Table 19, Table 20, Table 21, Table 22 and Table 23 further demonstrates that the reported H_2_ evolution rate should not be interpreted as an isolated descriptor of intrinsic photocatalytic activity. PCNNVs-L, TiO_2_/ZnIn_2_S_4_, Co_3_Se_4_/TiO_2_, and NiCoSe_2_/TiO_2_ exhibited H_2_ evolution rates of approximately 10,300, 6030, 6065, and 4976 μmol g^−1^ h^−1^, respectively. However, these values were obtained under conditions involving sacrificial electron donors, Xe-lamp irradiation, and, in some cases, Pt cocatalysts. In contrast, CoTiO_3_/g-C_3_N_4_ produced approximately 118 μmol g^−1^ h^−1^ under visible-light irradiation in pure water without a sacrificial agent. Therefore, its lower numerical rate should not be interpreted as evidence of intrinsically inferior performance, since the reaction involves the substantially more demanding process of unassisted water oxidation. These comparisons show that differences of one or more orders of magnitude may arise from the reaction medium, cocatalyst incorporation, irradiation conditions, and normalization basis, in addition to the structural and electronic properties of the semiconductor.

The influence of the reaction medium is also evident when the photocatalyst composition remains unchanged. For Pt-loaded g-C_3_N_4_, H_2_ evolution rates of approximately 114, 810, and 427 μmol g^−1^ h^−1^ were reported using ethanol, triethanolamine, and lactic acid, respectively. The more than sevenfold difference between the ethanol- and triethanolamine-assisted systems demonstrates that hole-scavenging kinetics, molecular adsorption, and interfacial oxidation pathways can influence the measured activity as strongly as photocatalyst composition. Similarly, pH-dependent results indicate that no universal optimum exists across semiconductor families because pH simultaneously modifies proton availability, surface charge, band-edge positions, cocatalyst activity, colloidal stability, and corrosion behavior. Consequently, photocatalytic H_2_ evolution should be interpreted within a coupled material–reactor–operation framework in which crystallinity, morphology, bandgap, catalyst loading, sacrificial-agent chemistry, pH, photon flux, and mass transfer jointly determine the observed response.

Reactor configuration and irradiation management are particularly important within this framework. Conventional systems commonly employ externally illuminated glass reactors with non-optimized geometries, which may generate substantial optical losses through scattering, reflection, attenuation, and non-uniform photon distribution. More advanced designs use quartz vessels, microreactors, thin-layer configurations, internal illumination, or solar-integrated systems to reduce optical path length and improve the spatial distribution of incident radiation. Such strategies are essential because photon limitation remains a major bottleneck in photocatalytic H_2_ production [149,150,161]. Nevertheless, improved reactor geometry does not automatically guarantee higher efficiency; the benefit depends on whether the illuminated region effectively overlaps with the catalytically active volume and whether mass-transfer limitations are simultaneously controlled.

Photocatalyst deployment introduces an additional trade-off. Suspended particles remain widely used because they provide a highly accessible surface area and intimate contact with the liquid phase. However, they also require downstream separation and may suffer from sedimentation, aggregation, and excessive light scattering at high concentrations. Immobilized catalysts and photocatalytic membranes simplify material recovery and may improve operational stability, but they can reduce the available active surface and introduce limitations associated with light penetration and the transport of reactants and products [149,162,163]. Thus, the selection between slurry and immobilized configurations should be based on a balance among photonic efficiency, mass transfer, catalyst recovery, and long-term operation rather than on catalyst activity alone.

A further limitation concerns the incomplete characterization of irradiation conditions. Many studies report only the nominal electrical power of the lamp without specifying the irradiance at the reactor surface, spectral distribution, illuminated area, source–reactor distance, or optical filter characteristics. This practice prevents reliable differentiation between intrinsic photocatalyst activity and reactor-dependent photonic effects. Complete optical reporting should therefore include incident irradiance, spectral range, photon flux, illuminated area, filter type, and AM 1.5G calibration when simulated solar irradiation is used [100,149,151]. The same level of rigor is required for reaction-atmosphere control and gas analysis. Closed systems, inert-gas recirculation, and calibrated H_2_ quantification reduce oxygen contamination, minimize uncertainty in kinetic measurements, and improve the reliability of stability tests conducted under prolonged irradiation [100,149,161].

From a technological perspective, the highest mass-normalized H_2_ evolution rate does not necessarily correspond to the most relevant or scalable system. Sacrificial-agent-assisted experiments are useful for evaluating charge separation and HER kinetics, but they do not represent stoichiometric overall water splitting and may conceal the energetic penalty associated with the oxidation half-reaction. Future benchmarking should therefore combine H_2_ evolution rates with apparent quantum yield, solar-to-hydrogen efficiency, volumetric and area-based productivity, energy consumption, long-term activity retention, and post-reaction structural characterization. Standardized reactor and irradiation conditions are also required to distinguish genuine improvements in semiconductor performance from enhancements caused primarily by favorable experimental conditions.

Current research is progressively moving toward modular reactors, continuous-flow systems, photocatalytic membranes, and integration with solar-harvesting technologies, thereby narrowing the gap between laboratory evaluation and pilot-scale application [151,161,163]. In this context, the reactor should not be regarded merely as a container for the photocatalyst but as an active component that governs photon delivery, species transport, thermal stability, gas recovery, and overall process feasibility. The most promising photocatalytic systems will therefore be those in which semiconductor design, reactor engineering, and operating conditions are optimized as an integrated platform.

### 5.1. Reactor Scale-Up and Large-Scale Applications

Based on the comparative analysis conducted in this review, the transition from laboratory-scale photoreactors to higher-capacity systems cannot be achieved through a proportional increase in reactor volume. Scale-up simultaneously modifies radiation distribution, optical path length, mass transfer, suspension mixing, gaseous-product separation, and thermal control. Consequently, a larger reactor volume does not necessarily ensure higher productivity or improved photocatalytic efficiency.

One of the main limitations is radiation attenuation within the reaction medium. As reactor depth increases, absorption, scattering, and shadowing may generate regions with limited photon availability. Under these conditions, part of the photocatalyst may remain insufficiently irradiated, whereas regions close to the light source may experience excessive irradiation, localized heating, or increased recombination losses. Therefore, the design of larger systems should prioritize short optical paths and a spatially uniform irradiation distribution.

Mass-transfer efficiency may also decrease during scale-up. Increasing the reaction volume makes it more difficult to maintain a homogeneous particle suspension, promotes sedimentation, and may generate concentration, temperature, and pH gradients. In addition, the accumulation of hydrogen and oxygen bubbles on the photocatalyst surface or reactor walls may partially block active sites and modify radiation propagation. Stirring, recirculation, and flow conditions should therefore be designed together with the optical geometry of the reactor.

Another relevant challenge is product separation and recovery. In higher-capacity systems, the simultaneous accumulation of hydrogen and oxygen may increase operational risks and promote backward reactions. Scalable designs should therefore incorporate continuous gas extraction, physical product separation, pressure control, leakage detection, and online monitoring of gas composition. These measures are essential for ensuring operational safety and improving the accuracy of production balances.

Scale-up also increases the energy consumption associated with pumps, stirring, cooling, recirculation, instrumentation, and gas-separation systems. Therefore, a high hydrogen production rate alone does not demonstrate technological feasibility. The assessment should also consider auxiliary energy consumption, photocatalyst stability, material recovery, maintenance requirements, and construction and operating costs.

A technically favorable pathway is the use of modular configurations with a short optical depth instead of indefinitely increasing the size of a single vessel. Panel reactors, thin-layer systems, continuous-flow units, recirculating circuits, and reactors containing immobilized photocatalysts may facilitate irradiation control, mass transfer, and material recovery. Process capacity can be increased by connecting multiple similar units while maintaining comparable optical and hydrodynamic conditions among modules (Table 26).

To properly assess pilot-scale and higher-capacity systems, parameters other than the hydrogen evolution rate should be considered, including the illuminated area, effectively irradiated volume, optical depth, volumetric and area-based productivity, radiation-utilization efficiency, auxiliary energy consumption, long-term stability, and the recovery efficiency of both gases and the photocatalyst. The combined use of these indicators makes it possible to distinguish a genuine increase in process capacity from a purely geometric enlargement of the reactor.

From an engineering perspective, the development of large-scale applications requires the integration of semiconductor design, reactor geometry, radiation distribution, hydrodynamics, safety, and energy and economic assessment. Scale-up should therefore be addressed as an integrated optimization process rather than as a subsequent and independent stage of photocatalyst development.

### 5.2. Techno-Economic Considerations of Photoreactor Configurations for Hydrogen Production

The viability of photocatalytic hydrogen production systems depends not only on the activity of the photocatalyst but also on other factors. It is also important to consider the reactor construction and installation costs, the energy consumption of auxiliary equipment, photocatalyst recovery or replacement, component maintenance, and storage of the produced gas.

Recent studies have shown that reactor design has a considerable influence on hydrogen production costs. Pinaud et al. analyzed four reactor configurations for a centralized plant: a single-bed particle suspension system, a dual-bed particle suspension system, a fixed-panel array, and a tracking solar concentrator array. Each module was designed to produce 1 t of H_2_ per day, whereas the levelized cost of hydrogen (LCOH) was calculated for a total production capacity of 10 t per day and a delivery pressure of 300 psig. The estimated direct capital costs were approximately USD 1.07, 1.69, 9.58, and 3.45 million for the single-bed, dual-bed, fixed-panel, and tracking concentrator systems, respectively. The corresponding hydrogen production costs were USD 1.60, 3.20, 10.40, and 4.10 kg^−1^ H_2_. In this analysis, particle-based reactors exhibited lower costs than panel-based systems. However, the single-bed configuration requires the separation of an H_2_/O_2_ gas mixture, thereby increasing both energy requirements and safety concerns [164].

Panel photoreactors facilitate photocatalyst reuse and avoid the need for downstream solid–liquid separation. However, they require reproducible fabrication procedures and uniform, stable photocatalyst coatings. They may also experience film delamination and provide a smaller accessible photoactive surface area. In contrast, slurry photoreactors offer a higher specific surface area and improved mass transfer; nevertheless, they may be affected by particle sedimentation and aggregation, together with additional costs associated with photocatalyst separation and recycling [165]. Gunawan et al. noted that large-scale panel fabrication can be complex and costly, whereas producing photocatalysts in powder form is comparatively straightforward. However, the economic advantage of either configuration depends on achieving a sufficiently high solar-to-hydrogen (STH) efficiency.

A recent study proposed a photocatalytic raceway reactor comprising two reaction volumes connected through a Z-scheme [166]. In this configuration, the nanoparticles responsible for the hydrogen evolution reaction (HER) and oxygen evolution reaction (OER) are suspended in separate compartments, thereby reducing the risk of forming explosive H_2_/O_2_ mixtures. The uninstalled reactor cost was estimated at USD 7.14 m^−2^, which is considerably lower than the costs reported for fixed-panel systems. Electricity is mainly required to operate the control systems and to purify and compress the produced H_2_ to approximately 30 bar. The estimated electricity consumption was 3.6 kWh kg^−1^ H_2_ for a plant producing 1 t day^−1^ and 2.5 kWh kg^−1^ H_2_ for a plant producing 50 t day^−1^.

## 6. Conclusions

Taken together, this review demonstrates that semiconductor-based photocatalytic hydrogen production should be analyzed as a coupled material–reactor–experimental condition system, rather than solely as an intrinsic property of the photocatalyst. Although the design of new materials, heterojunctions, defects, and cocatalysts remains essential to improve charge separation and surface kinetics, the observed efficiency depends directly on reactor geometry, irradiation distribution, optical path length, catalyst concentration, pH, type of sacrificial agent, reaction atmosphere, stirring, and thermal control.

The literature analysis reveals that one of the main current limitations in the field is the lack of standardization in the description of experimental conditions. Many studies report only the H_2_ production rate or the nominal lamp power, without providing sufficient information on the actual irradiance, illuminated area, spectral distribution, irradiated volume, catalyst loading, gas purging, medium composition, or quantification strategy. This omission hinders objective comparison among photocatalysts and may lead to misleading interpretations, where higher apparent activity is attributed to the material when, in fact, it may be influenced by differences in reactor design or operating conditions.

From a scientific perspective, this review confirms that the most promising systems will not necessarily be those with the most advanced photocatalyst but rather those in which material design is integrated with appropriate reactor engineering. Reactors with improved light distribution, lower optical attenuation, better mass-transfer control, efficient purging, reliable gas quantification, and stable thermal conditions will allow a more accurate evaluation of the real activity of the materials. Likewise, the use of metrics such as AQE, EQE, or STH should accompany H_2_ production rates in order to assess the energetic and photonic efficiency of the process.

Therefore, the main contribution of this review is to reorganize a field dominated by material-centered studies toward a more comprehensive perspective, in which the reactor and experimental conditions acquire the same level of importance as the semiconductor. This perspective is fundamental to improving reproducibility, establishing more rigorous comparison criteria, and advancing from laboratory-scale tests toward more scalable and reliable photocatalytic systems closer to real applications in solar hydrogen production.

## Figures and Tables

**Table 1:** Main thematic domains identified during the initial literature search.

Thematic Domain	Typical Focus in Retrieved Studies	Relevance to This Review	Interpretation
Photocatalyst synthesis	New materials, nanostructures, doping, morphology	Indirect	Useful when linked to H_2_ tests
Defect and band engineering	Vacancies, heterojunctions, charge transfer	Indirect to moderate	Relevant if experimental conditions are reported
Hydrogen evolution performance	H_2_ rate, HER activity, catalyst comparison	High	Core performance literature
Photocatalytic water splitting	Overall or assisted H_2_ production	High	Central thematic area
Photoelectrochemical systems	Electrodes, bias-assisted systems, PEC cells	Low to moderate	Often outside slurry reactor focus
Pollutant degradation photocatalysis	Dye removal, wastewater, oxidation studies	Low	Peripheral to present objective
Reactor and process conditions	Reactor setup, irradiation, pH, stirring, purging	Very high	Core analytical focus of this review

**Table 2:** Preliminary interpretation of the keyword co-occurrence network obtained from the initial search.

Observed Keyword Group	Representative Terms	Preliminary Interpretation	Relevance to Review
Core hydrogen production cluster	Hydrogen production, photocatalytic activity, HER	Central performance-oriented literature	Very high
Material design cluster	Layered semiconductors, nanosheets, graphitic carbon nitride	Focus on photocatalyst development	Moderate to high
Surface/interface cluster	Oxygen vacancies, co-catalysts, charge transfer, kinetics	Mechanistic optimization emphasis	Moderate
Operational conditions cluster	Irradiation, sacrificial agents, visible light	Experimentally relevant variables	High
Peripheral overlap cluster	Electrocatalysis, PEC water splitting, solar fuels	Adjacent but partially outside scope	Low to moderate

**Table 3:** Main sources of thematic noise identified during the initial search.

Type of Noise	Why It Appears in the Search	Why It Was Not Central to This Review	Filtering Rationale
Photoelectrochemical studies	Shared terms such as water splitting and HER	Different reactor and charge transport architecture	Limited direct comparability
Electrocatalysis literature	Common hydrogen evolution terminology	Not driven by suspended photocatalytic systems	Outside review focus
Pollutant degradation papers	Shared photocatalysis vocabulary	H_2_ not primary output variable	Peripheral application area
Pure material synthesis studies	Semiconductor and catalyst-related keywords	Often insufficient operational detail	Low reactor relevance
Optical/mechanistic-only papers	Focus on charge transfer or band structure only	Limited experimental configuration analysis	Indirect support only

**Table 5:** Key photoelectronic and interfacial processes governing semiconductor photocatalysis.

Stage	Phenomenon	Representative Timescale	Impact on H_2_ Production	Ref.
Absorption	Photon absorption and electron–hole pair generation	fs–ps	Determines the initial population of photogenerated charge carriers; strong absorption alone does not guarantee efficient charge utilization	[22]
Relaxation	Hot-carrier thermalization and trapping in defect states	fs–ps	Causes energy dissipation and may either promote charge separation or generate inactive recombination centers	[18]
Separation	Spatial separation of electrons and holes in the bulk or at semiconductor interfaces	ps–μs	Increases the probability that photogenerated carriers reach different active sites and participate in surface redox reactions	[19]
Recombination	Bulk, surface, trap-assisted, or interfacial electron–hole recombination	ps–s, system-dependent	Reduces the population of charge carriers available for HER and OER, thereby limiting H_2_ production	[22]
Transport	Migration or diffusion of charge carriers toward the surface or cocatalyst	ns–ms, system-dependent	Determines whether separated carriers can reach catalytically active sites before recombination	[18]
Surface reaction	Interfacial charge transfer followed by HER and OER	μs–s or longer	Directly governs proton reduction, water oxidation, and the formation of molecular H_2_ and O_2_	[20]

**Table 6:** Strategies to enhance charge separation and interfacial utilization of photogenerated carriers in photocatalytic systems.

Strategy	Mechanism	Advantage	Limitation	Ref.
Type-II heterojunction	A staggered band alignment drives photogenerated electrons and holes toward different semiconductors.	Promotes spatial charge separation, reduces direct recombination, and may combine complementary light-absorption properties.	The transferred carriers may retain weaker reduction and oxidation potentials. Suitable band alignment and intimate interfacial contact are therefore required.	[26]
Z-scheme	Low-energy electrons and holes recombine selectively, preserving strongly reducing electrons and strongly oxidizing holes.	Combines effective charge separation with the strong redox potentials required for HER and OER.	Requires efficient interfacial charge transfer. Poor contact, backward reactions, or instability of the transfer pathway may reduce performance.	[19]
S-scheme	Interfacial charge redistribution, band bending, and an internal electric field promote selective recombination of low-energy carriers.	Preserves high-energy electrons and holes while promoting directional charge separation and transfer.	The mechanism requires experimental evidence of charge redistribution, band bending, and transfer direction; band-position diagrams alone are insufficient.	[27]
Co-catalyst loading	Co-catalysts extract photogenerated electrons or holes and provide active surface sites for HER or OER.	Improves interfacial charge transfer, lowers kinetic barriers, and suppresses charge accumulation on the semiconductor surface.	Excessive loading may block light, cover active sites, promote aggregation, or increase cost, particularly when noble metals are used.	[18]
Defect engineering	Controlled vacancies, dopants, and surface defects modify electronic states, carrier trapping, adsorption, and charge-transfer pathways.	May extend light absorption, introduce active sites, improve adsorption, and promote charge separation when defects are controlled.	Excessive or deep defects may become nonradiative recombination centers and reduce the structural or chemical stability of the photocatalyst.	[22]
Nanostructuring	Control of particle size, morphology, dimensionality, porosity, and exposed facets shortens carrier-diffusion distances.	Increases accessible surface area and facilitates carrier migration toward active reaction sites.	Very small or highly defective structures may exhibit aggregation, surface recombination, photocorrosion, or excessive light scattering.	[26]

**Table 7:** Effect of reactor geometry on photocatalytic performance.

Geometry	Advantage	Limitation	Impact on H_2_ Production	Ref.
Batch reactor	Simple operation and easy implementation	Non-uniform light distribution	May lead to low reproducibility	[28]
Annular reactor	More uniform irradiation around the reaction zone	Moderate scaling complexity	Can improve photocatalytic efficiency	[29]
Flat-panel reactor	Low optical depth and improved light penetration	Limited reaction volume	High photon utilization efficiency	[32]
Microreactor	High surface-area-to-volume ratio	Low throughput	High apparent photocatalytic efficiency	[28]
Fixed-bed reactor	Easier catalyst separation and recovery	Limited illuminated surface area	Moderate H_2_ yield depending on light access	[29]
Continuous-flow reactor	Better scalability and process control	More complex experimental setup	Closer to practical application conditions	[32]

**Table 8:** Key geometric parameters in photocatalytic reactors.

Parameter	Importance	Ref.
Irradiated volume	Determines photon density within the reaction medium	[28]
Illuminated area	Controls the total area available for light absorption	[32]
Optical path length	Influences light attenuation and penetration depth	[29]
Lamp distance	Regulates irradiance reaching the reactor surface	[28]
Reactor geometry	Defines light distribution and hydrodynamic behavior	[32]
Light position	Affects irradiation uniformity across the reaction zone	[29]
Mixing	Enhances catalyst suspension, mass transfer, and exposure to photons	[28]

**Table 9:** Representative pH-dependent observations reported for particulate photocatalytic water-splitting systems.

pH Condition	Source-Supported Observation	Photocatalytic Implication	Interpretative Limitation	Ref.
Acidic, pH 3.5	A Z-scheme suspension composed of Ru/SrTiO_3_:Rh and BiVO_4_ exhibited pH-dependent water-splitting activity, with the highest reported activity at pH 3.5.	At this pH, aggregation and suitable contact between the particulate photocatalysts favored interparticle electron transfer.	The optimum was specific to the composition, redox pathway, and aggregation behavior of this Z-scheme system and should not be generalized to all photocatalysts.	[19]
Near-neutral, pH 6.8	Particulate photocatalyst sheets were reported to perform overall water splitting in pure water under near-neutral conditions at pH 6.8.	This result demonstrates that efficient overall water splitting can be achieved under near-neutral conditions when the material architecture and interfacial charge-transfer pathway are appropriately designed.	Operation at pH 6.8 does not establish near-neutral pH as universally optimal; performance remains dependent on the semiconductor, cocatalyst, and reaction system.	[19]
System-dependent	Chalcogenide cocatalysts can lower kinetic barriers and overpotentials associated with photocatalytic H_2_ evolution and promote interfacial charge utilization.	Favorable reaction conditions depend on the interaction among the semiconductor, chalcogenide cocatalyst, surface reaction pathway, and reaction medium.	The cited review does not establish a universal acidic, neutral, or alkaline optimum applicable to all chalcogenide-cocatalyst systems.	[21]

*Note:* The reported pH values correspond to representative systems described in the cited references and should not be interpreted as universal thresholds. Direct comparison requires equivalent photocatalyst composition, cocatalyst, electrolyte, irradiation, reactor configuration, and analytical conditions.

**Table 10:** pH-related thermodynamic, interfacial, and stability factors affecting photocatalytic H_2_ evolution.

Mechanism	Source-Supported Description	Implication for H_2_ Evolution	Interpretative Consideration	Ref.
Redox-potential and band-edge relationship	Changes in pH modify the thermodynamic potentials of the aqueous HER and OER redox couples and may alter their energetic relationship with the semiconductor band edges.	Changes the thermodynamic driving force available for proton reduction and water oxidation.	The magnitude of the pH response is material- and interface-dependent. Therefore, the solution pH and the potential reference scale should be reported.	[18,19]
Particle aggregation and interparticle contact	In particulate Z-scheme suspensions, pH-dependent aggregation can modify the physical contact between the hydrogen- and oxygen-evolution photocatalysts.	Suitable particle contact may facilitate interparticle electron transfer and improve overall water-splitting activity.	The effect is specific to the composition, surface properties, mediator, and aggregation behavior of the particulate system.	[19]
Interfacial adsorption and surface environment	The reaction medium influences the interaction of water, protons, sacrificial donors, intermediates, and products with active sites on the semiconductor surface.	Changes in surface adsorption can alter interfacial charge transfer and the availability of species involved in HER and OER.	Observed changes in H_2_production cannot be attributed to intrinsic semiconductor activity unless the medium composition and pH are controlled.	[18]
Photocorrosion and chemical stability	Some semiconductor photocatalysts, particularly chalcogenide-based materials, may undergo surface oxidation, dissolution, or structural modification during photocatalytic operation.	Material degradation can reduce the number of active sites, modify charge-transfer pathways, and decrease long-term H_2_ production.	Stability should be evaluated under clearly defined pH, irradiation, reaction-time, and medium conditions rather than inferred from short-term activity alone.	[18,21]
Cocatalyst-mediated surface kinetics	Chalcogenide cocatalysts can lower reaction-energy barriers and overpotentials and facilitate the utilization of photogenerated carriers at catalytic surface sites.	Improves interfacial reaction kinetics and may enhance the rate of photocatalytic H_2_ evolution.	The cited literature does not establish a universal pH optimum for all cocatalysts; the response depends on the semiconductor–cocatalyst combination and reaction medium.	[21]

*Note:* The effects summarized in this table are system-dependent and should not be interpreted as universal pH trends. Meaningful comparison requires reporting the photocatalyst and cocatalyst composition, initial and final pH, electrolyte or buffer composition, sacrificial agent, irradiation conditions, reactor configuration, and reaction time.

**Table 11:** Comparison of irradiation sources used in photocatalytic H_2_ production and their principal experimental implications.

Irradiation Source	Spectral and Operational Characteristics	Main Advantage	Main Limitation	Recommended Reporting Information	Ref.
Xe lamp	Broadband artificial irradiation commonly used with optical filters to evaluate photocatalysts under simulated solar or selected visible-light conditions.	Allows photocatalytic systems to be evaluated over a broad spectral range and can be adapted using cutoff or solar-simulation filters.	Nominal electrical power does not represent the irradiance reaching the reaction medium. Lamp heating and spectral differences from natural sunlight may also affect the experiment.	Lamp power, spectral distribution, filter type, irradiance at the reactor, illuminated area, and source–reactor distance should be reported.	[19]
Hg lamp	Artificial source characterized by intense ultraviolet emission and commonly employed to excite wide-bandgap semiconductor photocatalysts.	Provides sufficient photon energy for the direct excitation of UV-responsive materials such as TiO_2_ and related semiconductors.	Its spectrum is not representative of natural solar irradiation, and lamp-induced heating may influence reaction temperature and gas solubility.	The principal emission wavelength, nominal power, irradiance at the sample, reactor material, and temperature-control method should be specified.	[18]
AM 1.5G solar simulator	Artificial irradiation system intended to reproduce a standardized terrestrial solar spectrum under controlled laboratory conditions.	Facilitates comparison among photocatalytic systems evaluated under nominally equivalent simulated-solar conditions.	Reliable comparison requires calibration because lamp aging, spectral mismatch, non-uniform illumination, and differences in irradiance may modify the measured performance.	The simulator class, calibration method, irradiance, spectral range, illuminated area, and test duration should be reported.	[20]
Natural sunlight	Direct use of solar irradiation under outdoor operating conditions without an artificial optical energy source.	Provides a realistic evaluation of photocatalytic performance under environmental solar conditions and is relevant for scale-up studies.	Solar irradiance and spectral distribution vary with time, weather, geographical location, season, and angle of incidence, reducing experimental reproducibility.	Geographical location, date, exposure period, solar irradiance, illuminated area, reactor orientation, and meteorological conditions should be documented.	[19,20]

*Note:* Nominal lamp power alone is insufficient to compare photocatalytic H_2_ production systems. Meaningful comparison requires reporting the spectral distribution, irradiance at the reaction zone, illuminated area, optical filters, source–reactor distance, irradiation time, and reactor geometry.

**Table 12:** Optical parameters that should be reported in photocatalytic H_2_ production studies.

Parameter	Importance	Ref.
Light source type	Defines the emission spectrum and excitation conditions	[19]
Nominal power	Provides information on the electrical input of the irradiation system	[18]
Irradiance at the sample (mW cm^−2^)	Indicates the real incident energy reaching the reaction zone	[22]
Spectral distribution	Determines whether the emitted photons can activate the semiconductor bandgap	[27]
Lamp–reactor distance	Controls the intensity and spatial distribution of incident radiation	[28]
Illuminated area	Defines the effective area exposed to irradiation and photon flux density	[32]
Optical filters	Regulate the UV/visible fraction reaching the photocatalytic system	[20]
Irradiation time	Determines cumulative photon exposure and total H_2_ production	[19]

**Table 23:** Comparison of irradiation sources used in photocatalytic studies.

Irradiation Source	Spectral Region/Filter	Reported Condition	Typical Application	Main Advantage	Critical Limitation	Reference
Hg lamp	UV, 290–390 nm	125 W	Ramipril degradation using TiO_2_–HC and ZnO–HC	High UV emission; suitable for wide-bandgap semiconductors	Generates heat and may modify the reaction temperature	[124]
Xe lamp + visible-light cutoff filter	λ>420 nm	250 W	Cr(VI) reduction using TiO_2_–HC or SnO_2_–HC	Allows evaluation of photocatalytic activity under visible light	Does not represent the full solar spectrum; filter type and irradiation distance must be reported	[125]
Xe lamp	Simulated solar or visible light, depending on configuration	300 W	Tetracycline degradation using BiVO_4_–HC	Continuous emission; useful for approximating solar irradiation	Nominal lamp power is not equivalent to the real irradiance reaching the catalyst	[124]
High-power Xe lamp	300–800 nm	1.5–2.5 kW	Metoprolol and thiacloprid degradation using Zn_5_(OH)_6_(CO_3_)_2_–HC	High photon flux; useful for intensive irradiation tests	High electrical consumption; difficult direct extrapolation to real solar operation	[126]
Visible LED	475 nm	50 W	Carbamazepine degradation using Fe_3_O_4_/BiOBr/HC	Selective irradiation; lower heating than Hg or Xe lamps	Evaluates only a narrow spectral region	[127]
Multispectral LED	380–780 nm	7 W	Rhodamine B degradation using HC/BiOBr/Bi_12_TiO_20_	Low energy consumption; covers a broad visible range	Lower intensity than Xe lamps; irradiance measurement is required	[128]
Tubular UV-LED reactor	367 nm	30 UV-LEDs; 42 W radiant flux; 111 W electrical power consumption	Continuous degradation of contaminants in a tubular reactor	Adjustable intensity; lower residual heat; compact geometry	Requires proper spatial light distribution and thermal control	[129]
Xe-based photofluidized bed reactor	Laterally concentrated light; bed-dependent light distribution	300 W Xe lamp; tested intensities up to 48.41 W cm^−2^	Photo-assisted reverse Boudouard reaction	Improves light–particle contact and optical penetration	If gas flow excessively expands the bed, part of the catalyst may move outside the illuminated zone	[130]
AM 1.5G solar simulator	Standard global solar spectrum	1000 W m^−2^, equivalent to 100 mW cm^−2^	Comparative tests under “1 sun” conditions	Standard condition for solar comparison	Requires calibration; does not fully reproduce real variations in weather, time, or location	[131]

**Table 24:** Quantitative comparison of representative semiconductor-based photocatalytic H_2_ evolution systems under different reactor configurations and operating conditions.

Ref.	Photocatalyst	Reaction Type	Reactor Configuration	Catalyst Mode	Volume (mL)	Loading (g L^−1^)	Sacrificial Agent	pH	Light Source/ Wavelength	Irradiance/ Filter	H_2_ Rate (μmol g^−1^ h^−1^)	Volumetric/ Area Productivity	AQE/ AQY (%) at λ
[41]	2.4 wt.% WS_2_–TiO_2_	Sacrificial-agent-assisted H_2_ evolution	Sealed glass reactor	Suspended powder (1 mg)	22.1	0.045 ^a^	Methanol, 10 vol.%; NaOH as alkaline additive	Basic; adjusted using 0.5 M NaOH	300 W Xe lamp	NR; AM 1.5 filter	321	NR	NR
[42]	TiO_2_/ZnIn_2_S_4_ (TZIS2)	Sacrificial-agent-assisted H_2_ evolution	Closed three-neck flask	Suspended powder (20 mg)	100	0.200 ^a^	TEOA, 10 vol.%	NR	300 W Xe lamp	NR	6030	NR	NR ^b^
[43]	1.5 wt.% CoTiO_3_/g-C_3_N_4_	Unassisted H_2_ evolution in pure water	Sealed and evacuated glass reactor (Labsolar VIII AG)	Suspended powder (50 mg)	80	0.625 ^a^	None	6; adjusted using 0.2 M HCl	300 W Xe lamp, with or without a visible-light cutoff filter	NR; λ>400 nm cutoff for the visible-light test	118 (λ>400 nm); 330 (full UV–Vis spectrum)	NR	NR ^b^
[44]	25 wt.% Co_3_Se_4_/TiO_2_	Sacrificial-agent-assisted H_2_ evolution	Sealed and evacuated Labsolar-III AG reactor	Suspended powder (20 mg), magnetically stirred	NR	NR	TEOA, 20 vol.%	12	300 W Xe lamp	NR	6065	NR	1.8 at 365 nm
[45]	5 wt.% NiCoSe_2_/TiO_2_	Sacrificial-agent-assisted H_2_ evolution	Sealed Labsolar-III AG reactor	Suspended powder (20 mg)	NR	NR	TEOA, 20 vol.%	NR	300 W Xe lamp	NR	4976	NR	NR
[46]	Pt-loaded PCNNVs-L	Sacrificial-agent-assisted H_2_ evolution	Top-irradiation reactor connected to a glass gas-circulation system (CEL-PAEM-D8)	Suspended powder in an aqueous Pt/TEOA medium	45	0.556	TEOA, 5 mL	NR	300 W Xe lamp	NR; AM 1.5G simulated solar spectrum	10300	NR	3.5 at 420 nm; 1.8 at 550 nm; 1.2 at 650 nm
[87]	Pt-loaded C_3_N_4_-U (urea-derived)	Sacrificial-agent-assisted H_2_ evolution	Sealed glass reactor with a quartz lid, cooling jacket, and He purging	Suspended powder with Pt	100	NR ^c^	TEOA, 10 vol.%	6.3	SUNTEST CPS+ solar simulator with a 600 W Xe lamp	NR; filter ID 65 (λ>320 nm)	810	NR	NR
[87]	Pt-loaded C_3_N_4_-U (urea-derived)	Sacrificial-agent-assisted H_2_ evolution	Sealed glass reactor with a quartz lid, cooling jacket, and He purging	Suspended powder with Pt	100	NR ^c^	Lactic acid, 10 vol.%	6.3	SUNTEST CPS+ solar simulator with a 600 W Xe lamp	NR; filter ID 65 (λ>320 nm)	427	NR	NR
[87]	Pt-loaded C_3_N_4_-U (urea-derived)	Sacrificial-agent-assisted H_2_ evolution	Sealed glass reactor with a quartz lid, cooling jacket, and He purging	Suspended powder with Pt	100	NR ^c^	Ethanol, 10 vol.%	6.3	SUNTEST CPS+ solar simulator with a 600 W Xe lamp	NR; filter ID 65 (λ>320 nm)	114	NR	NR
[99]	MoS_2_/ α-Fe_2_O_3_ nanoheterojunction (MF-3)	Sacrificial-agent-assisted H_2_ evolution	Glass reactor	Suspended powder (20 mg)	50	0.400 ^a^	Na_2_SO_3_/Na_2_S (0.25/0.35 M); lactic acid, methanol, TEOA, and ethanol were also evaluated	Optimum at pH 7	350 W Xe lamp	NR; UV cutoff filter (λ>420 nm)	871.2 under the optimized conditions	NR	NR

*Notes:* ^a^ Catalyst loading was calculated from the reported catalyst mass and working volume. ^b^ The irradiation wavelength was identified, but the corresponding AQE/AQY percentage was not available in the extracted data. ^c^ The extracted information lists “2 g”; the original study should be consulted to determine whether this value corresponds to the total catalyst mass or to a catalyst loading of 2 g L^−1^. NR: not reported. TEOA: triethanolamine. AQE: apparent quantum efficiency. AQY: apparent quantum yield. The H_2_ evolution rates are presented on a mass-normalized basis, as reported in the original studies or calculated from the available experimental information. Direct ranking of intrinsic photocatalytic activity should be avoided because the studies employed different reaction media, irradiation spectra, reactor geometries, catalyst loadings, and normalization procedures.

**Table 25:** Comparative analysis of key reactor-related variables in photocatalytic H_2_ production studies using semiconductor materials.

Category	Variable	Conventional Approaches	Advanced/ Optimized Approaches	Main Advantage	Implications for Performance and Scale-Up	Reference
Reactor configuration	Reactor mode	Batch slurry reactors	Continuous-flow, microreactor, and modular reactor systems	Improved control of residence time, irradiation, and mass transfer	Enables more reproducible operation and facilitates process intensification	[152,153]
Reactor material	Optical window and vessel material	Borosilicate glass or conventional glass reactors	Quartz reactors and UV-transparent windows	Higher photon transmission, especially in the UV region	Reduces optical losses and improves comparability of photocatalytic activity	[152,154]
Catalyst configuration	Photocatalyst deployment	Suspended powder catalysts	Immobilized films, coated supports, and photocatalytic membranes	Easier catalyst recovery and lower post-treatment requirements	Improves operational stability but may introduce mass-transfer limitations	[152,155]
Hydrodynamics	Mixing and mass transfer	Magnetic stirring in batch reactors	Controlled flow, recirculation, microfluidic channels, and optimized mixing	Better gas–liquid–solid contact and reduced concentration gradients	Enhances reproducibility and supports scalable reactor operation	[153,156]
Light management	Irradiation geometry	External lamp illumination without detailed optical control	Optimized light path, thin-layer reactors, internal illumination, and solar concentrators	Improved photon utilization and reduced light attenuation	Increases apparent quantum efficiency and reactor productivity	[152,157]
Photonic characterization	Light intensity and spectral reporting	Lamp power reported only as nominal watts	Irradiance, spectral distribution, optical filters, and AM 1.5G calibration reported	Allows more reliable comparison among studies	Reduces ambiguity in evaluating intrinsic photocatalytic performance	[154,158]
Gas management	Inert atmosphere and product collection	Static purging with N_2_ or Ar before irradiation	Closed-loop gas recirculation and online gas chromatography	Minimizes O_2_ contamination and improves H_2_ quantification	Enhances accuracy in kinetic analysis and long-term stability tests	[152,159]
Thermal control	Temperature regulation	Ambient-temperature operation with limited monitoring	Jacketed reactors, external cooling, and controlled photothermal operation	Distinguishes photocatalytic from photothermal contributions	Improves mechanistic interpretation and energy-efficiency assessment	[157,160]
Reaction medium	pH and electrolyte control	Initial pH adjustment with diluted acids or bases	Buffered electrolytes and monitoring of local/bulk pH	Stabilizes semiconductor/electrolyte interfaces	Avoids misleading interpretation of band-edge alignment and redox driving force	[104,109]
Scalability	Reactor size and integration	Laboratory-scale vials or small glass reactors	Scalable panel, membrane, flow, and integrated solar reactor systems	Higher surface-area-to-volume control and better solar capture	Moves photocatalytic H_2_ production closer to practical deployment	[152,159]

**Table 26:** Main challenges and technical pathways for photocatalytic reactor scale-up.

Aspect	Scale-Up Challenge	Proposed Technical Pathway
Radiation distribution	Increased attenuation, scattering, and formation of low-irradiance regions	Use of panels, thin layers, internal illumination, or modular configurations with short optical paths
Mass transfer	Concentration gradients, photocatalyst sedimentation, and reduced homogeneity	Controlled recirculation, optimized mixing, and combined hydrodynamic and reactor-geometry design
Gas removal	Bubble accumulation, active-site blocking, and possible backward reactions	Continuous extraction, product separation, and online monitoring of gas composition
Thermal control	Non-uniform heating and modification of kinetics and gas solubility	Use of thermal jackets, heat exchangers, and automatic temperature control
Photocatalyst recovery	Difficulty in separating suspended particles and potential material losses	Immobilization on supports, photocatalytic membranes, or separation and recirculation systems
Operational safety	Simultaneous formation of hydrogen–oxygen mixtures, leakage, and pressure increase	Physical product separation, gas sensors, pressure control, and safe operating procedures
Energy consumption and costs	Higher demand for pumping, stirring, cooling, instrumentation, and separation	Integrated process optimization and assessment of productivity relative to energy and operating costs
Capacity expansion	Loss of laboratory-scale optical and hydrodynamic conditions	Modular scale-up through the connection of geometrically similar units

## Data Availability

The original contributions presented in this study are included in the article. Further inquiries can be directed to the corresponding authors.

## Abbreviations

The following abbreviations are used in this manuscript:

AQY	Apparent quantum yield
AQE	Apparent quantum efficiency
CB	Conduction band
EQE	External quantum efficiency
GC	Gas chromatography
HER	Hydrogen evolution reaction
LED	Light-emitting diode
OER	Oxygen evolution reaction
PCET	Proton-coupled electron transfer
PEC	Photoelectrochemical
PRISMA	Preferred Reporting Items for Systematic Reviews and Meta-Analyses
PZC	Point of zero charge
RHE	Reversible hydrogen electrode
STH	Solar-to-hydrogen
TEOA	Triethanolamine
VB	Valence band
